# Transcriptome Analysis of *Arabidopsis thaliana* in Response to *Plasmodiophora brassicae* during Early Infection

**DOI:** 10.3389/fmicb.2017.00673

**Published:** 2017-04-24

**Authors:** Ying Zhao, Kai Bi, Zhixiao Gao, Tao Chen, Huiquan Liu, Jiatao Xie, Jiasen Cheng, Yanping Fu, Daohong Jiang

**Affiliations:** ^1^State Key Laboratory of Agricultural Microbiology, Huazhong Agricultural UniversityWuhan, China; ^2^The Provincial Key Lab of Plant Pathology of Hubei Province, College of Plant Science and Technology, Huazhong Agricultural UniversityWuhan, China; ^3^State Key Laboratory of Crop Stress Biology for Arid Areas, College of Plant Protection, Northwest Agriculture and Forestry UniversityYangling, China

**Keywords:** *Arabidopsis thaliana*, *Plasmodiophora brassicae*, clubroot, transcriptome

## Abstract

Clubroot disease is a serious threat to cruciferous plants worldwide, especially to oilseed rape. However, knowledge on pathogenic molecular mechanisms and host interaction is limited. We presume that the recognition between *Arabidopsis thaliana* and *Plasmodiophora brassicae* occurs at the early stage of infection and within a relatively short period. In this study, we demonstrated changes on gene expression and pathways in *A. thaliana* during early infection with *P. brassicae* using transcriptome analysis. We identified 1,903 and 1,359 DEGs at 24 and 48 h post-inoculation (hpi), respectively. Flavonoids and the lignin synthesis pathways were enhanced, glucosinolates, terpenoids, and proanthocyanidins accumulated and many hormonal- and receptor-kinase related genes were expressed, caused by *P. brassicae* infection during its early phase. Therefore, the early interaction between *A. thaliana* and *P. brassicae* plays an important role in the entire infection process. The results provide a new contribution to a better understanding of the interaction between host plants and *P. brassicae*, as well as the development of future measures for the prevention of clubroot.

## Introduction

*Plasmodiophora brassicae* Wor. is a soil-borne, obligate, and biotrophic pathogen that attacks cruciferous plants and causes clubroot, leading to significant yield losses (Dixon, 2009). Clubroot has been reported in more than 60 countries or regions around the world, and, in recent years, the disease has become increasingly serious. In Europe, North America, East Asia, and other regions, clubroot has become a major threat (Dixon, 2009; Chittem et al., 2014; Galdames et al., 2014; Strehlow et al., 2014; Wallenhammar et al., 2014; Strelkov et al., 2016).

The lifecycle of *P. brassicae* can be divided into two phases: root-hair infection and cortical infection (Naiki, 1987). In the root-hair infection phase, *P. brassicae* resting spores feel host plants in the soil, form primary zoospores winding to the surface of the host roots, invade the root hairs, and form primary plasmodia, which then develop into secondary zoosporangia clusters and secondary zoospores. In the cortical infection phase, the secondary zoospores released into the soil, either directly or through root hairs, infect cortex cells in the root or develop into secondary plasmodia. This leads to the production of many resting spores and root swelling in the host plant (Kageyama and Asano, 2009). In previous studies, the cortical infection stage was considered to be the most important. In recent years, some researchers have suggested that root-hair infection also plays an important role, although there is little evidence (Macfarlane, 1958; Siemens et al., 2002; Malinowski et al., 2012; McDonald et al., 2014). High-throughput sequencing technologies have been used to study the pathogenic process and pathogenesis of infection by *P. brassicae*. Researchers have mainly focused on the middle or late phases (cortical infection or later), meaning that only limited information about the early infection phase has been obtained.

*P. brassicae* cannot be artificially cultured and there is no effective genetic transformation system, with the exception of one study on the *hph/gfp* gene by Feng et al. (2013). Therefore, knowledge of pathogenic molecular mechanisms is limited. Microarray chips, two-dimensional electrophoresis and high-throughput sequencing technology have been used to study gene expression in the host plant in response to *P. brassicae* infection. Siemens et al. explored the gene expression of *A. thaliana* Col-0 inoculated with *P. brassicae* after 10 and 23 days using a microarray chip. They found that more than 1,000 host genes were differentially expressed in the infected roots vs. the control roots. Starch, sulfur and secondary metabolism, auxin and cytokinin synthesis, and the expression of transport-related genes changed significantly, whereas that of genes associated with defense and lignin synthesis did not. In addition, some flavonoid genes were also differentially expressed (Siemens et al., 2006). Devos et al. and Cao et al. discovered protein changes in *Arabidopsis* following inoculation with *P. brassicae* using two-dimensional electrophoresis (Devos et al., 2006; Cao et al., 2008). Devos et al. observed 35 up-regulated and 11 down-regulated proteins 4 days after *P. brassicae* inoculation, which were mainly associated with defense, cell metabolism, cell differentiation, and active oxygen activity (Devos et al., 2006). Cao et al. observed changes in the expression of 20 proteins (with 13 spots increasing and seven spots decreasing) 12, 24, and 48 h post-inoculation, including lignin synthesis, cytokinin synthesis, calcium steady-state, glycolysis, and oxygen activity (Cao et al., 2008). Agarwal et al. detected 147, 27, and 37 differentially expressed genes (DEGs) in *A. thaliana* after 4, 7, and 10 days, respectively, using a microarray chip. They further deduced that changes observed at 4 days post-infection (dpi) were mainly related to host and pathogen recognition and signal transduction (Agarwal et al., 2011). Using transcriptome analysis, Chen et al. observed major changes between sensitive and resistant varieties of *Brassica rapa* 0–96 h post-infection in metabolism, transport, and signal transduction (Chen et al., 2015). Nowadays, new high-throughput technologies are gradually being used to study the interaction between *P. brassicae* and the host, such as metabotyping, laser microdissection coupled to transcriptional profiling coupled and miRNA sequencing (Wagner et al., 2012; Schuller et al., 2014; Verma et al., 2014). The *P. brassicae* genome was recently sequenced, which will be very convenient for studies on the interaction between host plants and *P. brassicae* in the future (Schwelm et al., 2015; Rolfe et al., 2016).

Therefore, the response of *A. thaliana* during the early stages of infection with *P. brassicae* has not been enough studied. Furthermore, the number of *A. thaliana* genes that have been detected during the early stages of infection with *P. brassicae* is also limited. In order to clarify the early events taking place between the pathogen and the host, we analyzed differentially expressed genes and pathways in *A. thaliana* 24 and 48 h following infection with *P. brassicae*, using transcriptome analysis. In addition, the molecular mechanism of *A. thaliana*'s response to *P. brassicae* in the early infection phase is described.

## Materials and methods

### *P. brassicae* and plant material

The *P. brassicae* strain ZJ-1 was originally isolated from a diseased plant in a rapeseed field in Zhijiang County, Hubei Province, China. The resting spores were extracted and purified according to the method described by Castlebury et al. (1994). The *A. thaliana* ecotype Columbia (Col-0) was kindly donated by Dr. Yangdou Wei at the University of Saskatchewan and seven mutant lines (AT5G66590 mutant SALK_121768; AT3G04720 mutant SALK_082089C; AT1G50060 mutant SALK_033410; AT3G12500 mutant SALK_028588C; AT4G36000 mutant SALK_108883; AT2G19990 mutant SALK_014249C and AT1G73620 mutant SALK_100586C) used in this study were originally provided by the Nottingham Arabidopsis Stock Centre (NASC, Nottingham, UK). The plants were cultured and propagated in a plant-growth chamber [20 ± 1°C, 14/10 h light/dark (Wuhan Ruihua Instrument & Equipment Co. Ltd, Wuhan, China)].

### Microscopic observation of the infection process

To determine the important time-points during early infection, the infection process was investigated. Col-0 was sown in sterilized garden soil, and inoculated with *P. brassicae* (10^7^ spores/mL) following 30 days of growth in the chamber. Every 24 h after inoculation, the roots were gently washed three times using sterile water, dyed with trypan blue, then placed on a slide with distilled water and observed using an inverted microscope (Nikon Eclipse 80 I).

### Sample preparation

Col-0 seeds were sown in sterilized garden soil (Peilei Organic Fertilizer Co., Ltd., China) in a pot (4.5 × 4.5 × 5 cm). The seedlings were cultured in a plant-growth chamber for 30 days before inoculation. *P. brassicae* were inoculated into the plants as described by Siemens et al. (2009). Each *Arabidopsis* seedling was inoculated with 2 mL of the resting spore suspension (1 × 10^7^ spores/mL) and the inoculated seedlings were cultivated in the growth chamber. Sample roots were collected at three time-points (before inoculation, 24 and 48 h after inoculation), washed five times with sterile water, immediately ground into powder with liquid nitrogen, and saved in RNase-free Eppendorf tubes at −80°C for RNA isolation.

To ensure repeatability and to reduce error, we planted, inoculated, and collected the plants at 24 and 48 hpi three times, with 72 plants for each time-point. Triplicate RNA samples from each time-point were mixed for sequencing. The plants were sampled before inoculation, to act as a control (CK).

### RNA isolation

Total RNA samples were extracted with a TRIzol® Plus RNA Purification Kit (Takara, Dalian, China) and treated with RNase-free DNase I (Takara, Dalian, China) according to the manufacturer's instructions. The RNA quality was checked using a Nanodrop spectrophotometer (Thermo Fisher Scientific Inc., Wilmington, DE, USA).

### RNA-Seq sequencing

The cDNA library was prepared and sequenced by BGI (Beijing). mRNA was enriched using the oligo(dT) magnetic beads. The mRNA was disrupted to form short fragments (~200 bp) and first-strand cDNA was synthesized with a random hexamer-primer using the mRNA fragments as templates. Buffer, dNTPs, RNase H, and DNA polymerase I were added to synthesize the second strand. The double-stranded cDNA was purified with the QiaQuick PCR extraction kit and washed with EB buffer for end-repair and single nucleotide A (adenine) addition. Finally, sequencing adaptors were ligated to the fragments. The fragments were purified by agarose gel electrophoresis and enriched by PCR amplification. RNA-seq was performed using the Illumina HiSeq™ 2000 sequencing platform. The raw RNA-seq data have been deposited at SRA (http://www.ncbi.nlm.nih.gov/sra/) under accession number PRJNA348394 (https://www.ncbi.nlm.nih.gov/bioproject/348394).

### Digital gene expression analysis

Some sub sequences and low quality sequences were removed from the original data obtained from BGI to generate clean reads. The clean reads were then compared to the *A. thaliana* genome (TAIR 10) and reference gene sequences using the SOAP aligner software and SOAP 2 (Li et al., 2009). Finally, the sequence results were evaluated in terms of read quality, alignment, saturation, and the distribution of reads on reference genes (Wang et al., 2009). Gene expression was calculated by the number of reads mapped to the reference sequences using the RPKM method (Mortazavi et al., 2008).

Referring to the method described by Audic and Claverie (1997), the DEGs were selected with the following standards: FDR was < 0.001 and there was at least a two-fold change (>1 or < −1 in log2 ratio value) in RPKM between two samples (FDR ≤ 0.001 and |log2 Ratio| ≥ 1).

### Quantitative PCR analysis

To validate the RNA-seq results, we selected 40 DEGs for qRT-PCR analysis. For the qRT-PCR, 10-μL reaction systems were analyzed in triplicate using the CFX96 Real-Time PCR Detection System (Bio-Rad, USA). Each reaction mixture contained 5 μL of 1 × SYBR Green Supermix (Bio-Rad, USA), 1 μL of first-strand cDNA obtained from the same RNA samples mentioned above, 0.15 μL of forward primer and 0.15 μL of reverse primer (10 μmol/L). RNase/DNase-free water was added to the final volume. The qRT-PCR program was as follows: denaturation at 95°C for 2 min, followed by 49 amplification cycles of 95°C for 5 s, and 60°C for 30 s. A melting curve was generated to verify the specificity of the amplification (from 65 to 95°C with an increment of 0.5°C per cycle, with each cycle held for 5 s). Expression of *A. thaliana actin* (AT3G18780) was used to normalize the expression of the genes in each corresponding qRT-PCR sample using *actin*-specific primers (Table S1). The qRT-PCR assay was repeated at least twice for each gene, with three replicates.

### DEG analysis and annotation

The DEGs selected above were compared to the Kyoto Encyclopedia of Genes and Genomes (KEGG) pathway database (Kanehisa et al., 2008) and Gene Ontology (GO; Ashburner et al., 2000) for GO and pathway annotation. To obtain a more general overview of changes in gene expression, the DEGs were also analyzed with the MAPMAN program (Thimm et al., 2004).

### Flavonoid and proanthocyanidin determination

To investigate whether *P. brassicae* could induce flavonoid production in the early stage of infection, Col-0 seeds were sown in sterilized garden soil (Peilei Organic Fertilizer Co., Ltd., China) in a pot. The seedlings were cultured in a plant-growth chamber for 30 days before inoculation. Flavonoids in the CK, 24 and 48 hpi samples were detected with high-performance liquid chromatography (HPLC) following the method described by Päsold et al. (2010). Three kinds of flavonoid, quercetin, naringenin, and kaempferol (Sigma), were used as standards. Spectrophotometry was used to determine proanthocyanidin levels, using the Oligomeric Proanthocyanidins Kits (Shanghai Biotechnology Co., Ltd). The proanthocyanidins were examined at 500 nm. The experiments were repeated twice.

### Observation of clubroot symptoms

Col-0 and the seven *Arabidopsis* mutant lines were planted and inoculated as previously described. After inoculation, the *Arabidopsis* Col-0 and mutants were cultured in a plant-growth chamber. After 18 days, the plants were collected, washed five times using sterile water, and the clubroot symptoms were observed.

## Results

### The early process of *A. thaliana* infection by *P. brassicae*

Following the inoculation of *Arabidopsis* with resting spores, the early infection process was continuously observed every 24 h. At 24 hpi, many *P. brassicae* primary zoospores had been absorbed into the seedling roots, and a few primary zoospores began to infect the root hairs. At 48 hpi, most of the primary zoospores had begun to infect the root hairs and the primary plasmodia could be observed in some of the root hairs. The primary plasmodia could be seen in most of the root hairs at 72 hpi and the plasmodia grew and differentiated in the root hairs from 96 to 168 hpi. At 192 hpi, secondary zoosporangia of *P. brassicae* were clearly visible. At 216 hpi, the secondary zoospores began to infect the *Arabidopsis* cortex cells (Figure S1). The primary infection with *P. brassicae* zoospores occurred mainly between 24 and 48 hpi; at 72 hpi, infection with primary zoospores was complete and primary plasmodia had been formed. The results were consistent with those reported by Kageyama and Asano (2009) and Cao et al. (2008). Therefore, three time-points (0 hpi [CK], 24 hpi [24 h], and 48 hpi [48 h]) were selected for RNA-seq analysis.

### RNA-Seq analysis

Original reads were generated from RNA-seq by 50-bp paired-end sequencing. The impurities (such as sub sequences, low-quality reads, etc.) were removed first to gain clean reads for the analysis. The proportion of clean reads in the sequencing results of the three samples (CK, 24 and 48 h) was over 96% (98.19, 98.21, and 96.87%, respectively) and the sequencing depths were close to saturation (Figure S2). The clean reads of each sample were subsequently used to match the *Arabidopsis* TAIR 10 gene sequences (allowing for two mismatched bases). The qualified matched rates of the three samples were 94.07, 93.45, and 93.62%, respectively, and the total matched proportion reached 84% (84.96, 84.40, and 84.04%, respectively). These data are shown in Table S2. This showed that the sequencing results could be used for subsequent analyses.

### Analysis and verification of differentially expressed genes (DEGs)

**Figure 1:**
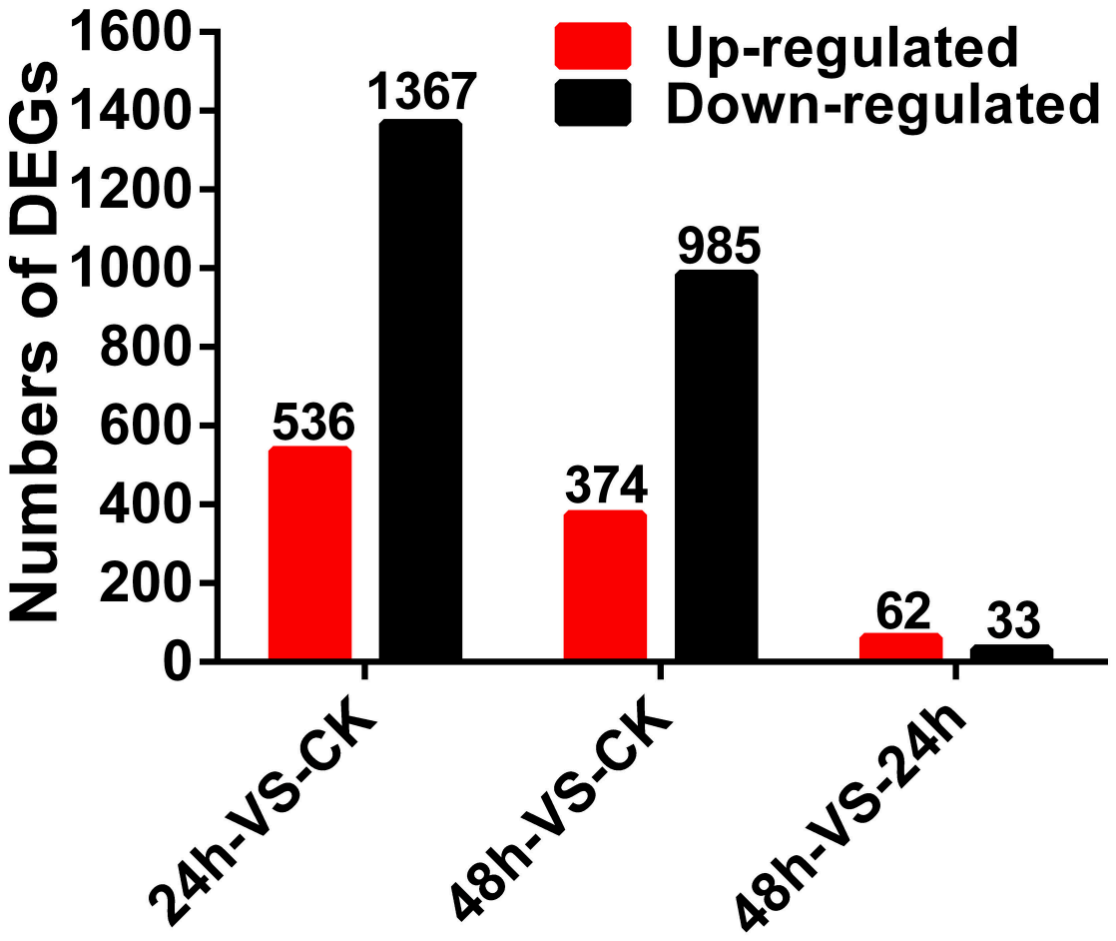
**Differentially expressed genes of *A. thaliana* during early infection by *P. brassicae***.

Gene expression levels were calculated using the RPKM method (Mortazavi et al., 2008) and the DEGs (FDR ≤ 0.001 and |log2 Ratio| ≥ 1) were counted according to the method described by Audic and Claverie (1997). Compared to the CK sample, 536 genes were up-regulated and 1,367 genes were down-regulated in the 24 h sample; 374 genes were up-regulated and 985 genes were down-regulated in the 48 h sample. In addition, compared with the 24 h sample, 62 genes were up-regulated, and 33 genes were down-regulated in the 48 h sample (Figure 1).

**Figure 2:**
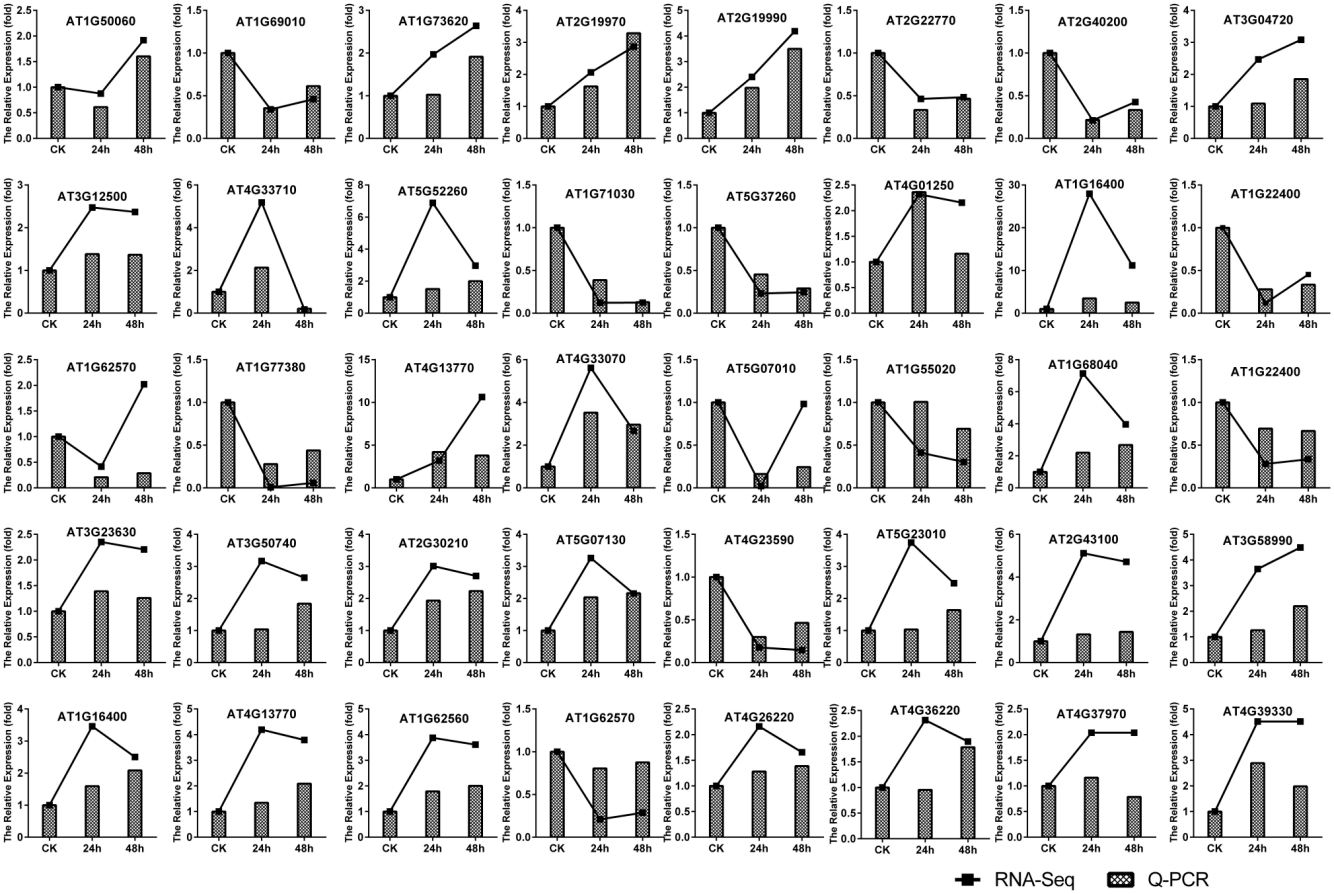
**Verification of gene expression patterns in *A. thaliana* during early infection by *P. brassicae* by qRT-PCR**. Forty DEGs (including 10 PR genes, three MYB transcription factor genes, one WRKY gene, 11 genes from the model of the IAA, SA, JA, and cytokinins signal pathway, 11 genes associated with secondary metabolism, and four cell wall-related genes) were selected to validate the reliability of the RNA-seq results. The expression of *A. thaliana actin* (AT3G18780) was used to normalize the expression of the genes in each corresponding qRT-PCR sample using actin-specific primers. The relative level of expression of individual genes in the CK was set as 1.0.

In order to confirm the results of the RNA-seq analysis, 40 DEGs were selected for qRT-PCR, including 10 PR genes, three MYB transcription factor genes, one WRKY gene, 11 genes involved in the IAA, SA, JA, and cytokinin signaling pathways, 11 genes associated with secondary metabolism and four genes related to the cell wall (Table S1). The results indicated that all 40 DEGs followed similar expression patterns as those observed by RNA-seq (Figure 2). This further confirms the high degree of reliability of the RNA-seq method used in this study.

### Functional annotation analysis of DEGs

**Figure 3:**
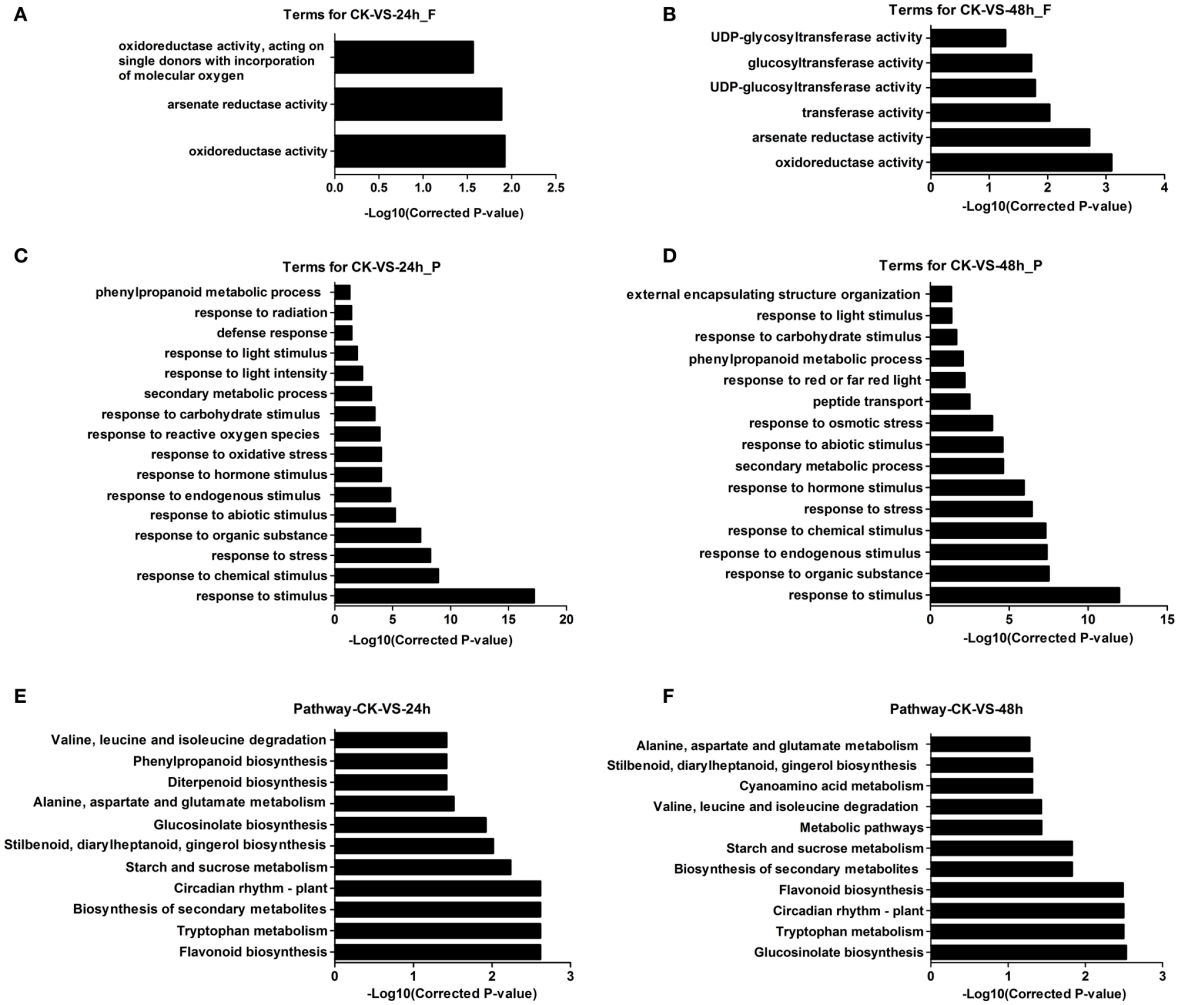
**GO and pathway analyses of DEGs in *A. thaliana* during early infection by *P. brassicae*. (A,B)** show the function analysis result, **(C,D)** show the process result and, **(E,F)** show pathway results.

GO has three ontologies: molecular function, cellular component, and biological process. The DEGs were used for the GO enrichment analysis. The results showed that the significantly enriched GO terms (*P* ≤ 0.05) were in the molecular function and biological process ontologies, while no significant terms were found in the cellular component ontology (Table S3). There were 1,095 DEGs at 24 hpi and 771 DEGs at 48 hpi enriched in the molecular function ontology. The category “oxidoreductase activity” was significantly annotated in both the 24 and 48 hpi samples. Additionally, “glucosyltransferase activity” was annotated at 48 hpi (Figures 3A,B, Table S3). In the biological processes ontology, 1,054 and 767 DEGs could be enriched, with 16 and 15 significantly enriched GO terms at 24 and 48 hpi, respectively. The main enriched terms were “response to stimuli” (including chemical, biological, hormones, endogenous, and carbohydrate), “response to stress” (including oxidative, reactive oxygen species), “secondary metabolic process” and “phenylalanine metabolic process” (Figures 3C,D, Table S3).

Proteins interact with each other to function in certain biological activities. Pathway analysis could be used to further understand the biological function of genes. The differentially expressed gene sequences were mapped to the reference canonical pathways in KEGG. A total of 921 and 659 DEGs were annotated in the KEGG database and assigned to 111 and 106 KEGG pathways at 24 and 48 hpi, respectively. The significantly enriched pathways (*P* ≤ 0.05) were 24 and 19 at 24 and 48 hpi, respectively (Table S4). At 24 hpi, the most common term was “flavonoid biosynthesis,” followed by multiple metabolism and biosynthesis pathways, such as “tryptophan metabolism,” “secondary metabolism,” and “glucosinolate biosynthesis.” These pathways were also enriched at 48 hpi. The “circadian rhythm–plant” pathway was also enriched (Figures 3E,F, Table S4).

### Biotic stress overview pathway analysis

**Figure 4:**
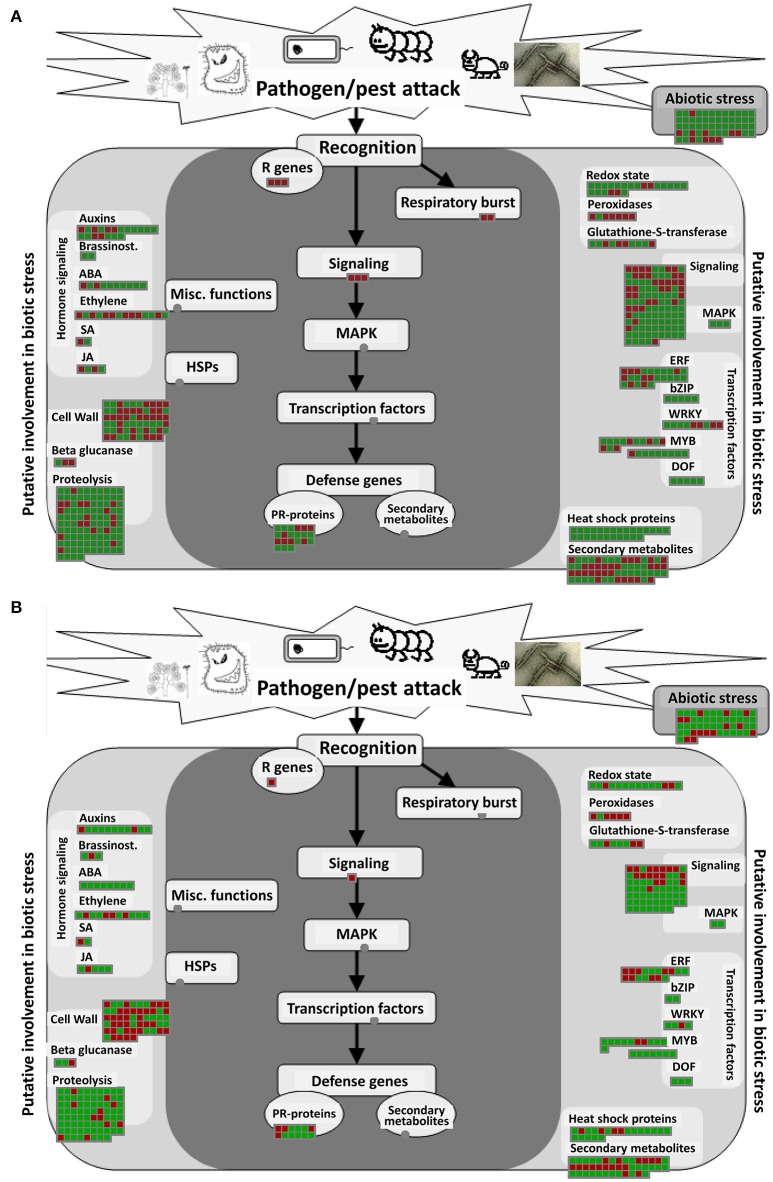
**Biotic stress pathway analyses of DEGs in *A. thaliana* during early infection by *P. brassicae***. Analysis of the biotic stress pathway of DEGs was performed using MAPMAN software. Red boxes mean up-regulated genes and green mean down-regulated genes. **(A)** 24 h after inoculation; **(B)** 48 h after inoculation. The pathway frames are from the MAPMAN software database.

The biotic stress pathway includes a series of DEGs induced by pathogens, insects, and other organisms that infect their hosts. DEGs involved in the response of *A. thaliana* to infection by *P. brassicae* would be greatly reflected in this pathway. Through MAPMAN analysis, 596 DEGs at 24 hpi and 438 DEGs at 48 hpi were found to be altered in biotic stress pathways, with similar components in the two samples. In the pathway overview, eight parts were included: “secondary metabolism,” “hormones,” “PR proteins,” “signal process,” “transcription factors,” “cell wall-related genes,” “protein degradation process-related genes,” and “heat shock proteins” (Figure 4).

### Metabolism overview and flavonoid pathway analysis

**Figure 5:**
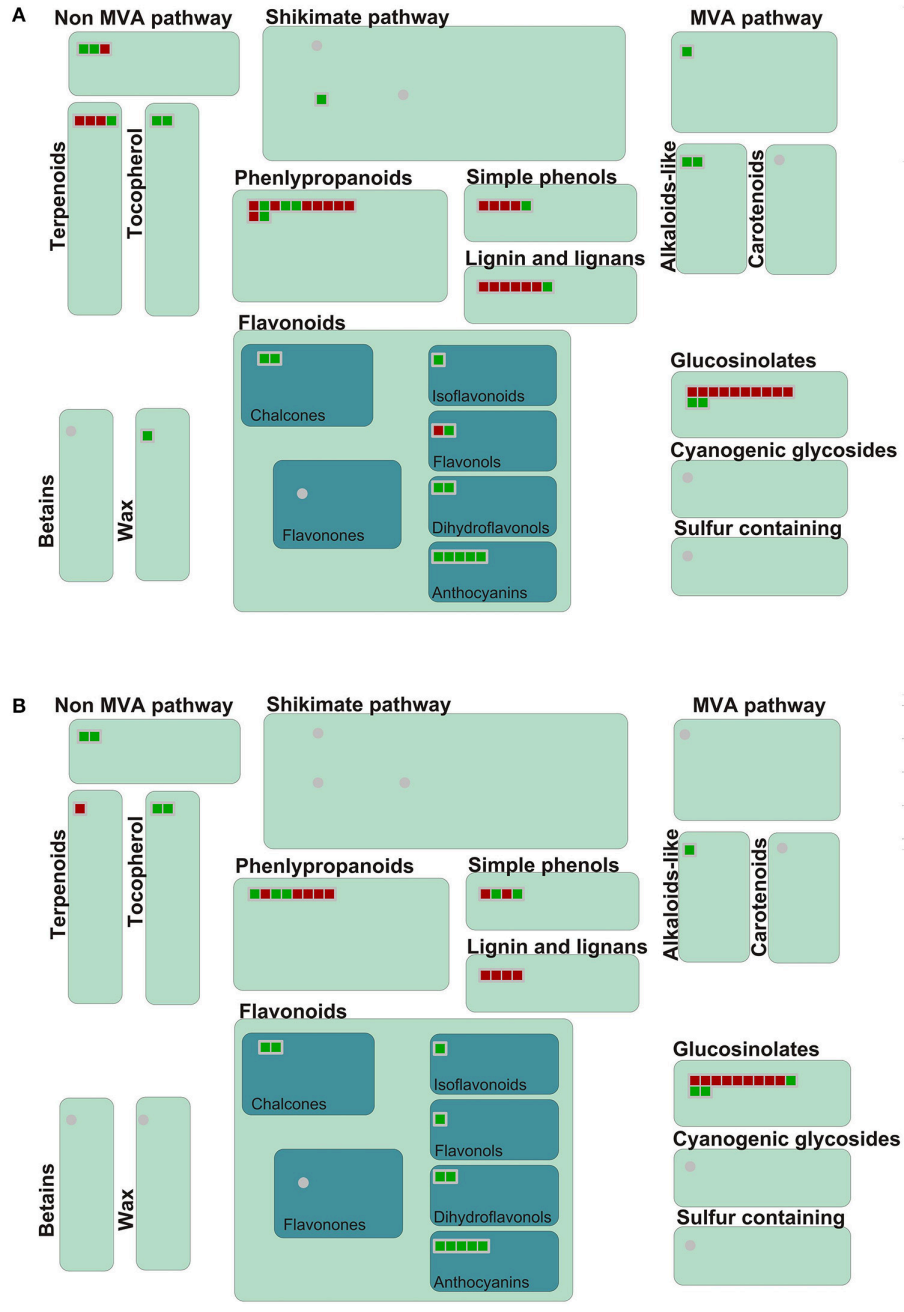
**Secondary metabolism pathway analyses of DEGs in *A. thaliana* during early infection by *P. brassicae***. Secondary metabolism pathway analysis of the DEGs was performed using MAPMAN software. Red boxes mean up-regulated genes and green mean down-regulated genes. **(A)** 24 h after inoculation; **(B)** 48 h after inoculation. The pathway frames are from the MAPMAN software database.

In GO and KEGG pathway analysis, metabolism-related genes were found to have changed significantly following inoculation with *P. brassicae*. Therefore, an overview of metabolism and secondary metabolic pathways was conducted according to MAPMAN analysis (Figure 5, Figure S3). In metabolism overview pathways, 252 DEGs at 24 hpi and 194 DEGs at 48 hpi were concentrated, mainly in “cell wall,” “lipids,” and “secondary metabolic” pathways (Figure S3). In the secondary metabolism pathway, the expression of genes involved in the biosynthesis of five compounds (phenlypropanoids, lignin, glucosinolates, terpenoids, and simple phenols) was obviously increased at 24 and 48 hpi (Figure 5).

In MAPMAN, the secondary metabolism and flavonoid pathways, which were the most significantly enriched pathways in the KEGG analysis, were also visibly changed. As shown in the flavonoid biosynthesis pathway map, 33 DEGs distributed across the whole flavonoid biosynthesis pathway at 24 hpi and 25 DEGs at 48 hpi were activated (Figure S4).

**Figure 6:**
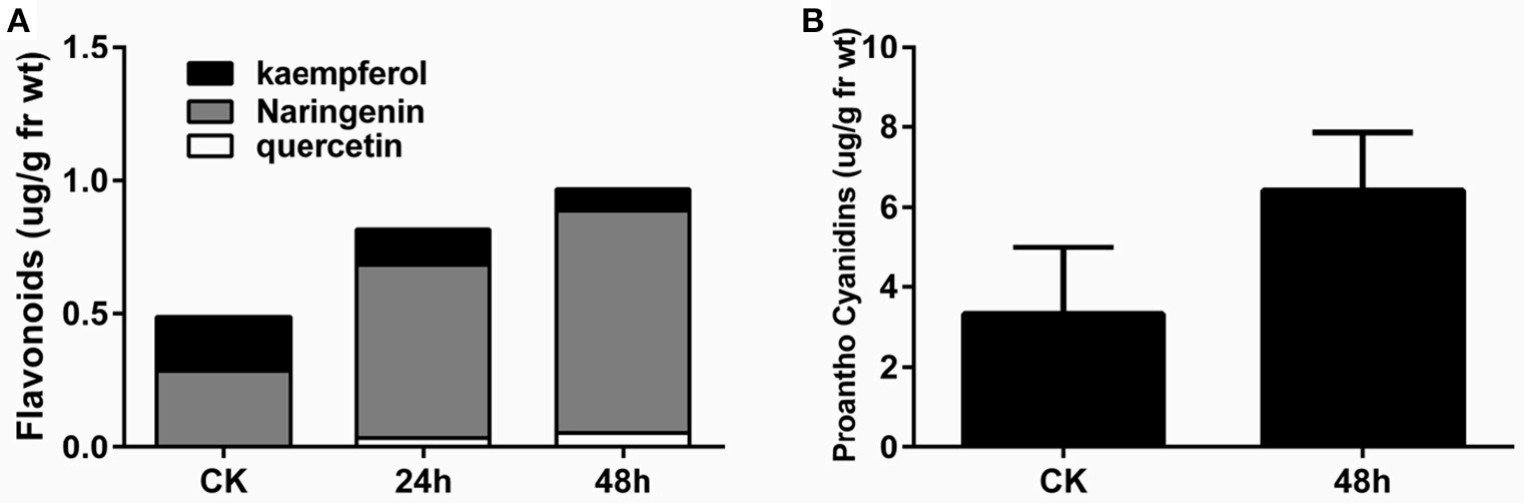
**Flavonoid (A)** and proanthocyanidin **(B)** contents in *A. thaliana* roots during early infection by *P. brassicae*.

In order to validate the increase in flavonoids at the early stage of *P. brassicae* infection, the flavonoid content (quercetin, naringenin, and kaempferol) in the roots was determined by HPLC, as described by Päsold et al. (2010) (Figure 6A). Compared to the CK sample, naringenin, and quercetin were increased two-fold in the 24 and 48 hpi samples, while no changes in kaempferol content were observed. Compared with the control, proanthocyanidins increased approximately two-fold in the roots at 48 hpi, as monitored by the spectrophotometric method (Figure 6B).

### Lignin (phenylpropanoids) biosynthesis pathway analysis

**Figure 7:**
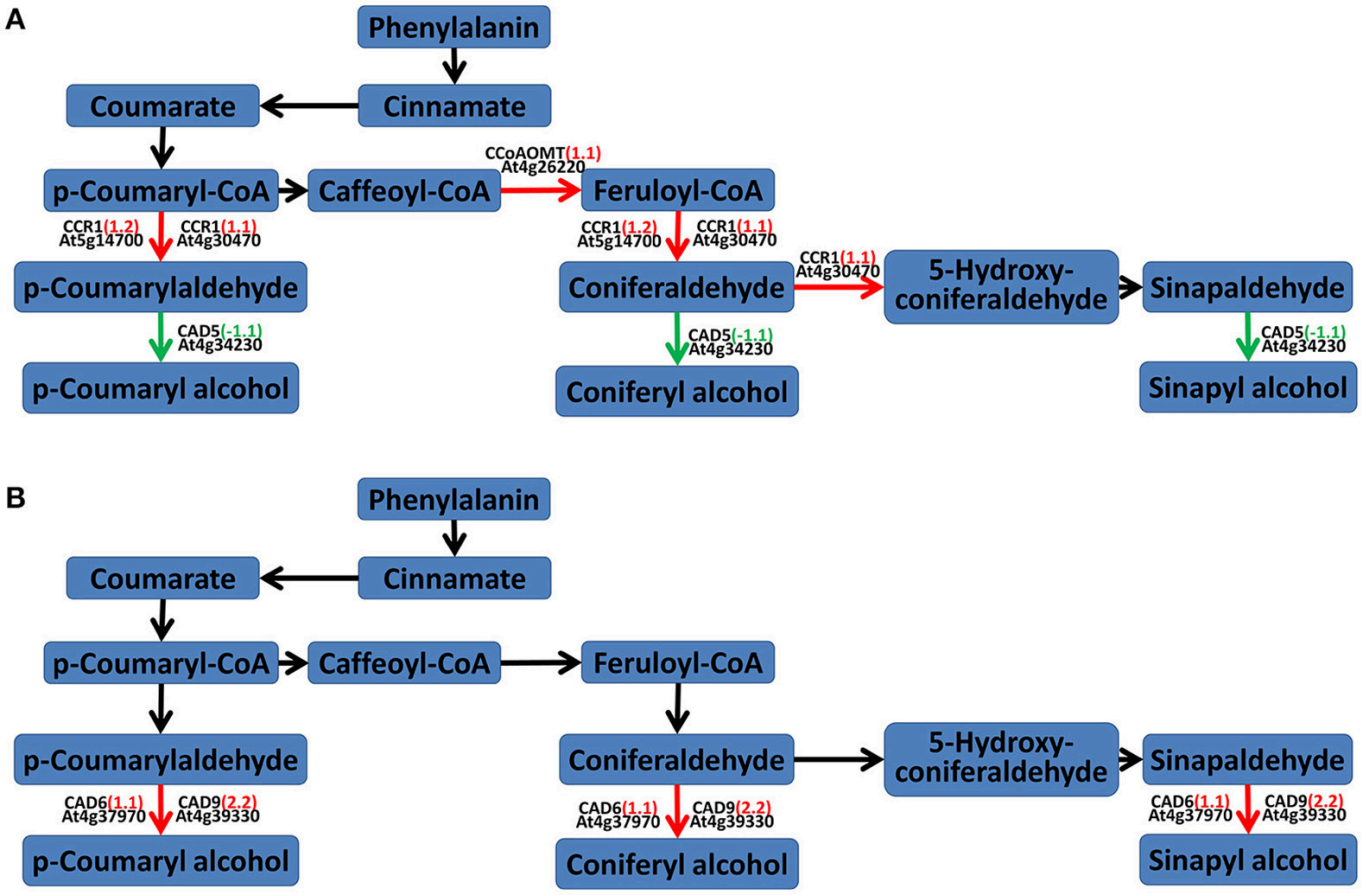
**Lignin pathway analyses of DEGs in *A. thaliana* during early infection by *P. brassicae***. Analysis of the lignin (phenylpropanoid biosynthesis) pathway of DEGs was performed using MAPMAN software. Red arrows mean up-regulated genes and green mean down-regulated. Black ones mean no change. **(A)** 24 h after inoculation; **(B)** 48 h after inoculation. The pathway frames are from the MAPMAN software database.

According to the results of the secondary metabolism pathway analysis in MAPMAN and KEGG, the lignin (phenylpropanoids) biosynthesis pathway was also changed. At 24 hpi, two cinnamyl-coenzyme A reductase (CCR1) genes (AT4G30470 and AT5G14700) and a putative caffeoyl-CoA O-methyltransferase (CCoAOMT) gene (AT4G26220) were up-regulated and a cinnamyl alcohol dehydrogenase (CAD) gene *CAD5* (AT4G34230) was down-regulated, which would lead to coumaryladehyde and coniferaldehyde accumulation. At 48 hpi, *CAD6* (AT4G37970) and *CAD9* (AT4G39330) were both up-regulated (Figure 7).

### Analysis of plant hormone signal pathway

**Figure 8:**
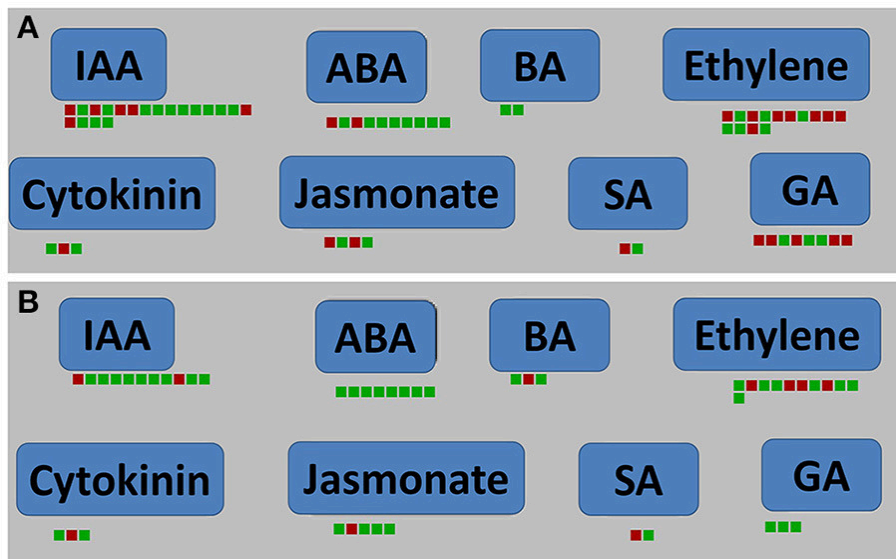
**Plant hormone signal pathway analyses of DEGs in *A. thaliana* during early infection by *P. brassicae***. Plant hormone signal pathway analyses were performed using MAPMAN software. Red boxes mean up-regulated genes and green mean down-regulated. **(A)** 24 h after inoculation; **(B)** 48 h after inoculation. The pathway frames are from the MAPMAN software database.

Infection with *P. brassicae* causes swelling in the host roots. Our present results indicate that the expression of some hormone-related genes changed significantly at the root-hair stage of *P. brassicae* infection of *A. thaliana*. 19 genes (including six up-regulated and 13 down-regulated) at 24 hpi and 11 genes (including two up-regulated and nine down-regulated) at 48 hpi were related to the IAA pathway (Figure 8). Twenty-five DEGs at 24 hpi and 20 DEGs at 48 hpi were involved in the tryptophan metabolism pathway by KEGG analysis, and were significantly focused on IAA (Figure S5). At both 24 and 48 hpi, one up-regulated gene (AT3G23630) and two down-regulated genes (AT1G22400 and AT5G05860) related to the cytokinin pathway, and 40 genes related to JA, ET, GA, ABA, and BA pathways at 24 hpi and 32 genes at 48 hpi were also changed (Figure 8, Table S5).

At 24 and 48 hpi, the S-adenosyl-L-methionine gene (AT1G68040) was up-regulated and the S-adenosylmethionine-dependent methyltransferase (AT5G37990) was down-regulated by MAPMAN analysis (Figure 8). PR proteins are important elements of SA-mediated signaling pathways. Analysis of biotic stress pathways (Figure 4) revealed 30 differentially expressed PR protein genes at 24 hpi and 22 at 48 hpi, respectively (Table S6).

**Figure 9:**
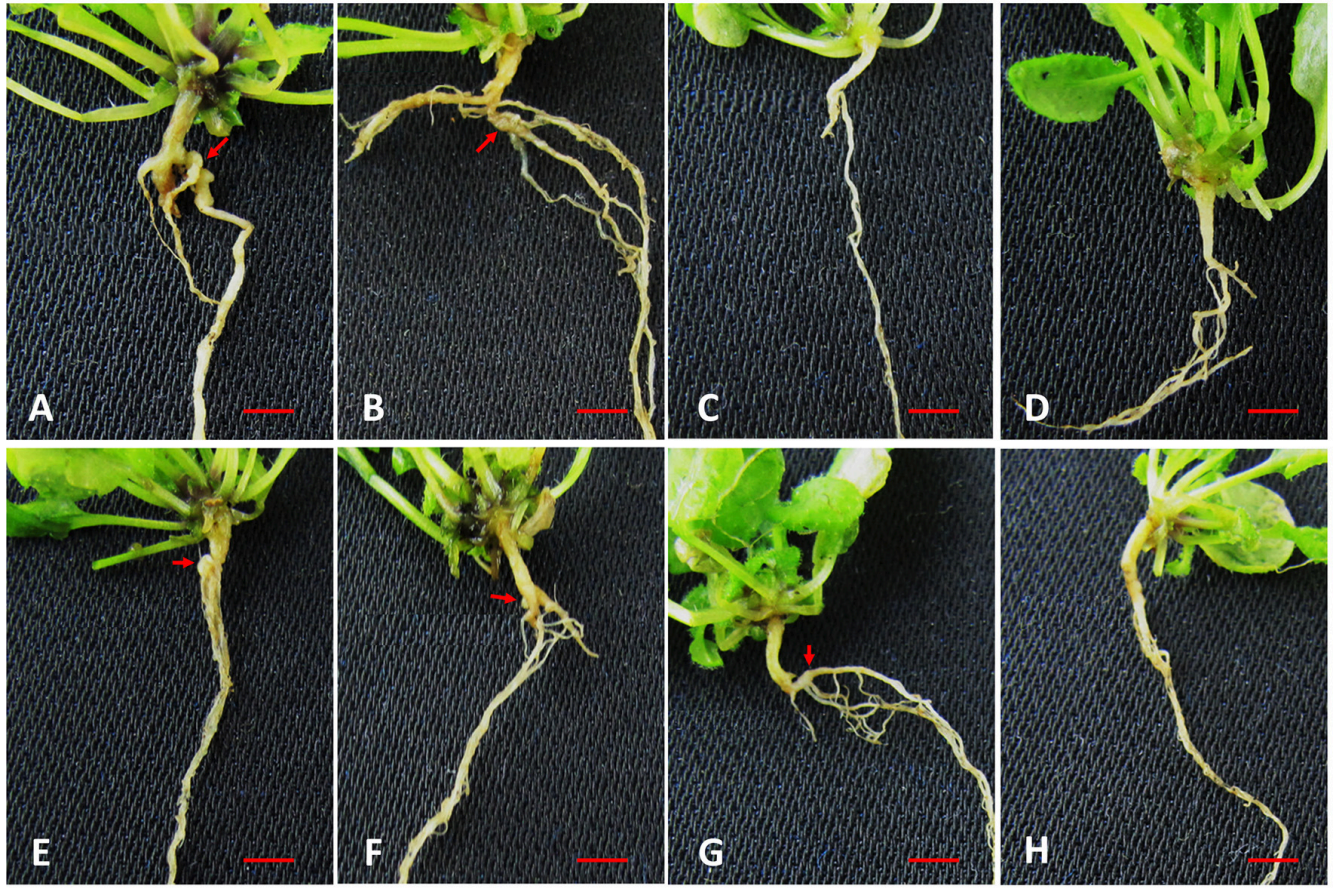
**The clubroot symptoms of *Arabidopsis* Col-0 and mutants infected by *P. brassicae***. Clubroot disease in *A. thaliana* ecotype Col-0 and mutants 18 days after inoculation with *P. brassicae*. **(A)** AT5G66590 mutant (SALK_121768); **(B)**AT3G04720 mutant (SALK_082089C); **(C)** AT1G50060 mutant (SALK_033410); **(D)** AT3G12500 mutant (SALK_028588C); **(E)** AT4G36000 mutant (SALK_108883); **(F)** AT2G19990 mutant (SALK_014249C); **(G)** AT1G73620 mutant (SALK_100586C); **(H)** Col-0. Bar = 0.5 cm.

In order to confirm the role played by PR proteins in resistance to *P. brassicae* infection, Col-0 and seven *Arabidopsis* PR gene mutants were inoculated. Compared with Col-0, at 18 days after inoculation (Figure 9H), two PR gene mutants (AT5G66590 and AT3G04720) displayed very severe clubroot symptoms (Figures 9A,B), three PR gene mutants (AT4G36000, AT2G19990, and AT1G73620) displayed mild clubroot symptoms (Figures 9E–G) and two PR gene mutants (AT1G50060 and AT3G12500) displayed no clubroot symptoms (Figures 9C,D).

### Analysis of other related pathways

The proteasome pathway targets proteins modified by ubiquitin molecules. It plays an important role in plant defense against pathogen infection. According to the MAPMAN analysis, the DEGs mainly encoded E3 ubiquitin-related RING and FBOX proteins (Figure S6). The expression of many heat shock proteins (including HSP70, HSP80, and HSP90) was also changed.

During pathogen infection, effectors are secreted to interfere with the defense response. Conversely, the host produces some receptors that combine with the effectors and inhibit pathogen invasion. We also found 32 receptor kinase genes changed at 24 hpi and 15 receptor kinase genes at 48 hpi (Figure S7).

## Discussion

Recognition between the pathogen and host is possible within a short time period and is not a long process. We presumed that *P. brassicae* and host recognition occurred during the root-hair infection phase. Therefore, we chose two early time-points (24 hpi: primary zoospore absorption, a few primary zoospores begin to infect the root hairs; 48 hpi: most of the primary zoospores have begun to infect the root hairs) to study, using the RNA-seq technique. A total of 1,903 and 1,359 DEGs at 24 and 48 hpi were detected by RNA-seq, respectively. We found that many genes and pathways, which have been reported during the late stage of *P. brassicae* infection (Siemens et al., 2006; Päsold et al., 2010), were active at the early infection stage. These results suggest that the early infection stage (24 and 48 hpi) is a key phase for the recognition and interaction between *A. thaliana* and *P. brassicae*. This is consistent with the hypothesis of McDonald et al., who stated that recognition between *P. brassicae* and its host, as well as successful cortex infection, may occur during the root-hair infection phase (McDonald et al., 2014).

### Response of the flavonoid biosynthesis pathway in *A. thaliana* during early infection by *P. brassicae*

Flavonoids are a group of secondary metabolites with many biological functions, including plant defense (Dixon, 2001). Siemens et al. (2006) carried out transcriptome analysis of the response of *A. thaliana* to *P. brassicae* at the late stage of infection (10 and 23 dpi), and reported that many flavonoid pathway genes seemed to be up-regulated. As a result, flavonoid accumulation was confirmed in clubroot galls. *A. thaliana* mutants associated with the flavonoid biosynthetic pathway were slightly altered in their response to clubroot, but flavonoid treatment cannot reduce gall development. This suggested that flavonoids might influence the transport of auxin into root galls (Päsold et al., 2010). Treatment with ProCa, an inhibitor of oxoglutaric acid-dependent dioxygenases, also reduced clubroot development (Päsold and Ludwig-Müller, 2013). Only one flavonol synthase gene (At5g08640) was up-regulated at 2 dpi, and a gene encoding dihydroflavonol-4-reductase (At4g27250) was down-regulated at 7 dpi in response to *P. brassicae* (Jubault et al., 2013). In the present study, we identified 32 DEGs distributed across the whole flavonoid biosynthesis pathway at 24 hpi, and 25 DEGs were activated at 48 hpi. The flavonoid contents (quercetin, naringenin, and kaempferol), which accumulated in the clubroot galls, significantly increased at 24 and 48 hpi, before the formation of the clubroot galls. Our results suggest that the flavonoid pathway is most likely involved in the response of *A. thaliana* to early infection with *P. brassicae*; it might have functions beyond the transport of auxin.

### Lignin biosynthesis in the response of *A. thaliana* to *P. brassicae* during early infection

Lignification is a mechanism that involves the enhancement of the cell wall in order to resist infection by foreign pathogens (Vance et al., 1980). Siemens et al. (2006) reported that some *A. thaliana* genes involved in cell division and expansion were up-regulated 10 and 23 days after *P. brassicae* inoculation.CCoAOMTwas suppressed at 48 hpi (Cao et al., 2008). The expression of *4CL, CCR1*, and *CAD5*, which encode enzymes involved in the lignin biosynthesis pathway, was suppressed 7 days after *P. brassicae* inoculation (Agarwal et al., 2011; Jubault et al., 2013). We found that in the early stage of infection by *P. brassicae, CCR1* and *CCoAOMT* were up-regulated and *CAD5* was down-regulated at 24 hpi, while *CAD6* and *CAD9* were up-regulated at 48 hpi. Following the activation of the lignin (phenylpropanoids) biosynthesis pathway, lignin precursors (coumarylalcohol, coniferyl alcohol, and sinapylalcohol) accumulated. At the same time, the expression of many cell-wall related genes was also enhanced (Figure 4, Figure S3). Lignification has been proved to induce systemic resistance in cucumber (Hammerschmidt and Kuć, 1982); Cell wall hydroxyproline enhancement and lignin deposition were reported to participate the resistance of cucumber to *Cladosporium cucumerinum* (Hammerschmidt et al., 1984); Cell wall-bound phenolic acid and lignin contents were related to the resistance of date palm to *Fusarium oxysporum* (El Modafar and El Boustani, 2001). Our results also suggest that lignin begins to accumulate in order to enhance resistance to *P. brassicae* at the early stage of infection.

### Hormonal changes in the response of *A. thaliana* to early infection with *P. brassicae*

Hormone-mediated signal transduction pathways play a very important role in the interactions between pathogens and plants. Previous research showed that *P. brassicae* stimulated the synthesis of host hormones (mainly auxin and cytokinins) (Ludwig-Müller et al., 2009; Malinowski et al., 2016), and they are essential for the development of root galls (Dekhuijzen and Overeem, 1971; Butcher et al., 1974; Jahn et al., 2013). The inhibition of auxin transport results in decreased clubroot symptoms in *B. rapa* (Devos and Prinsen, 2006). Many genes related to auxin and cytokinin synthesis, metabolism, and transport were found to be expressed at 10 and 23 dpi (Siemens et al., 2006), with only one auxin-responsive protein being down-regulated 4 days after *P. brassicae* inoculation (Agarwal et al., 2011). In the present study, 13 DEGs at 24 hpi and 11 DEGs at 48 hpi in the IAA pathway, as well as three genes in the cytokinin pathway, were found to be highly expressed. At the same time, a significant change in the tryptophan metabolism pathway was mainly concentrated on IAA synthesis in the early phase of *P. brassicae* infection. The results suggest that IAA and cytokinins are regulated at a very early stage of infection (24 hpi or earlier).

The roles of salicylic acid and jasmonic acid in the resistance of plants to disease, including clubroot, are well-known. Salicylic acid has been reported to suppress clubroot (Lovelock et al., 2013; Chen et al., 2016; Manoharan et al., 2016). Treatment with salicylic acid reduced the development of clubroot in *A. thaliana* and *B. napus* via the activation of several host defense-related pathways (Lahlali et al., 2013, 2014; Li et al., 2013; Lovelock et al., 2013). In our laboratory, we also found that the *Arabidopsis bik1* mutant exhibited strong resistance to *P. brassicae*, which was possibly mediated by SA-inducible mechanisms (Chen et al., 2016). In the present study, 30 and 22 PR protein genes were detected at 24 and 48 hpi, respectively. Seven up-regulated SA signal PR protein gene mutants of *Arabidopsis* were used to detect sensitivity to *P. brassicae*. Two mutants were highly sensitive and three were slightly sensitive to *P. brassicae*. In general, the resistance-related PR protein genes were activated at the early stage of infection. Furthermore, many ethylene-, GA-, ABA-, and JA-related genes were detected in our study. Thus, hormones contribute to the interaction between *A. thaliana* and *P. brassicae* at the very early phase of infection.

### Pathways for biosynthesis of other metabolites and ubiquitin in the response of *A. thaliana* to *P. brassicae* infection

Glucosinolates, a group of secondary metabolites from plants in the Brassicaceae family, have long been associated with clubroot disease symptoms and defense, along with auxin biosynthesis (Grubb and Abel, 2006; Ludwig-Müller, 2009). Previous studies have shown that the regulation of glucosinolate and IAA biosynthesis might differ in *Brassica* and *Arabidopsis* (Ludwig-Müller, 2009). In the present study, we found that the expression of genes related to glucosinolate and terpenoid biosynthesis were significantly increased in the metabolism pathway (Figure 5). In the flavonoid biosynthesis pathway, the biosynthesis of catechin and epicatechin increased, and these can combine to form proanthocyanidins. At the same time, proanthocyanidins were found to have significantly accumulated, based on spectrophotometric analysis at 48 hpi (Figure 6, Figure S4). To our knowledge, this is the first time that proanthocyanidins have been reported to have accumulated in response to *P. brassicae* infection. In addition, clear changes were observed in RING and FBOX proteins in the ubiquitin pathway, as well as in the heat shock proteins (Figure 4, Figure S6).

Proanthocyanidins carry out antioxidant, free radical-scavenging, anti-inflammatory, and anti-carcinogenic activities (Wang and Stoner, 2008). In medicine, they also exhibit antitumor effects and regulate signal transduction, promote tumor cell apoptosis, arrest the cell cycle, and inhibit angiogenesis. Glucosinolates and terpenoids exhibit resistant activity to cancer (Fahey et al., 1997; Wade et al., 2007; Lage et al., 2010). Some studies have found that heat shock proteins involved in the FBOX-RING ubiquitin-proteasome pathway are very closely linked to antitumor action (Whitesell and Lindquist, 2005). *P. brassicae* infects the host, multiplies in the host cells, leads to the enlargement of host root cells and eventually results in tumor generation, which is termed clubroot. This process is similar to that which occurs during tumor formation and growth in humans. Therefore, we presumed that *Arabidopsis* may recognize the infection and start producing related substances and activating the FBOX-RING ubiquitin-proteasome pathway to inhibit the reproduction of the pathogen and the formation of clubroot, in a similar manner to that of antitumor substances in humans.

### Receptor kinase in the response of *A. thaliana* to early infection by *P. brassicae*

In recent years, biotrophic and hemibiotrophic pathogens have been shown to secrete effectors to regulate hosts, while the hosts also produce kinase proteins (receptors) to resist pathogen infection (Jones and Dangl, 2006). The response of some receptor kinase genes to *P. brassicae* infection has been reported (Ueno et al., 2012; Hatakeyama et al., 2013). We also found that many receptor kinase genes are differentially expressed at 24 and 48 hpi (Figure S7). This confirms that the interaction between *A. thaliana* and *P. brassicae* might have already occurred during the early phase of *P. brassicae* infection.

We also noticed that defense pathways were not significantly induced, based on the one- or two-fold changes observed for almost all the genes listed. This could be for two reasons: first, infection did not progress well-enough within the first 48 h, and second, the *Arabidopsis* ecotype Col-0 is susceptible to clubroot. The defense pathways are always induced later and are weaker in susceptible hosts compared to resistant lines.

## Conclusion

In this study, we analyzed the *A. thaliana* transcriptome during the very early phase of *P. brassicae* infection (24 and 48 hpi). A total of 1,903 and 1,359 DEGs at 24 and 48 hpi, respectively, were identified. During this early phase, the flavonoid and lignin synthesis pathways were enhanced, glucosinolates, terpenoids, and proanthocyanidins accumulated and many hormone- and receptor kinase-related genes were differentially expressed in response to *P. brassicae* infection. Therefore, the early interaction between *Arabidopsis* and *P. brassicae* plays an important role in the entire infection process. These results provide a new basis for studying the interaction between the host and *P. brassicae*.

## Author contribution

YZ, TC, JX, JC, YF, and DJ designed research; YZ, KB, and ZG performed research; YZ, KB, and HL analyzed data; and YZ and DJ wrote the paper.

### Conflict of interest statement

The authors declare that the research was conducted in the absence of any commercial or financial relationships that could be construed as a potential conflict of interest.

## Supplementary material

Figure S1 **The early process of *A. thaliana* infection by *P. brassicae* stained with trypan blue**. The red arrows point to *P. brassicae* at different stages (1–2d, primary zoospore; 3–7d, primary plasmodial; 8d, zoosporangia; 9d, secondary zoospores; and 10–15d, secondary plasmodia).

Figure S2 **Evaluation of sequencing quality (A)** and saturation **(B)** analysis of DEGs in *A. thaliana* during early infection by *P. brassicae*.

Figure S3 **Metabolism pathway analyses of DEGs in *A. thaliana* during early infection by *P. brassicae***. Metabolism pathway analysis of the DEGs was performed using MAPMAN software. Red boxes mean up-regulated genes and green mean down-regulated genes. **(A)** 24 h after inoculation; **(B)** 48 h after inoculation. The pathway frames are from MAPMAN software database.

Figure S4 **Flavonoid biosynthesis pathway analyses of DEGs in *A. thaliana* during early infection by *P. brassicae***. Analysis of the flavonoid biosynthesis pathway of DEGs was performed. Red boxes mean up-regulated genes and green mean down-regulated genes. **(A)** 24 h after inoculation; **(B)** 48 h after inoculation.

Figure S5 **Tryptophan metabolism pathway analyses of DEGs in *A. thaliana* during early infection by *P. brassicae***. Analysis of the tryptophan metabolism pathway of DEGs was performed. Red boxes mean up-regulated genes and green mean down-regulated genes. **(A)** 24 h after inoculation; **(B)** 48 h after inoculation.

Figure S6 **Ubiquitin pathway analyses of DEGs in *A. thaliana* during early infection by *P. brassicae***. Analysis of the ubiquitin pathway of DEGs was performed using MAPMAN software. Red boxes mean up-regulated genes and green mean down-regulated genes. **(A)** 24 h after inoculation; **(B)** 48 h after inoculation. The pathway frames are from the MAPMAN software database.

Figure S7 **Receptor-like kinases gene analyses of DEGs in *A. thaliana* during early infection by *P. brassicae***. Analysis of the receptor-like kinases pathways of DEGs were performed using MAPMAN software. Red boxes mean up-regulated genes and green mean down-regulated genes. **(A)** 24 h after inoculation; **(B)** 48 h after inoculation. The pathway frames are from the MAPMAN software database.

Table S1 **The genes and primers used in qRT-PCR**.

Table S2 **The statistical results mapped to the genes of samples CK, 24 and 48 h**.

Table S3 **The significantly enriched GO terms of DEGs in *A. thaliana* during early infection by *P. brassicae* (*P* ≤ 0.05)**.

Table S4 **The significantly enriched pathways of DEGs in *A. thaliana* during early infection by *P. brassicae* (*P* ≤ 0.05)**.

Table S5 **Hormone regulation-related genes of DEGs in *A. thaliana* during early infection by *P. brassicae***.

Table S6 **PR proteins of DEGs in *A. thaliana* during early infection by *P. brassicae***.
