# Supplementary figures and images for: Transcriptome Analysis of Arabidopsis thaliana in Response to Plasmodiophora brassicae during Early Infection
Source: Front Microbiol. 2017 Apr 24;8:673. doi: 10.3389/fmicb.2017.00673 (PMC5401899; doi:10.3389/fmicb.2017.00673)

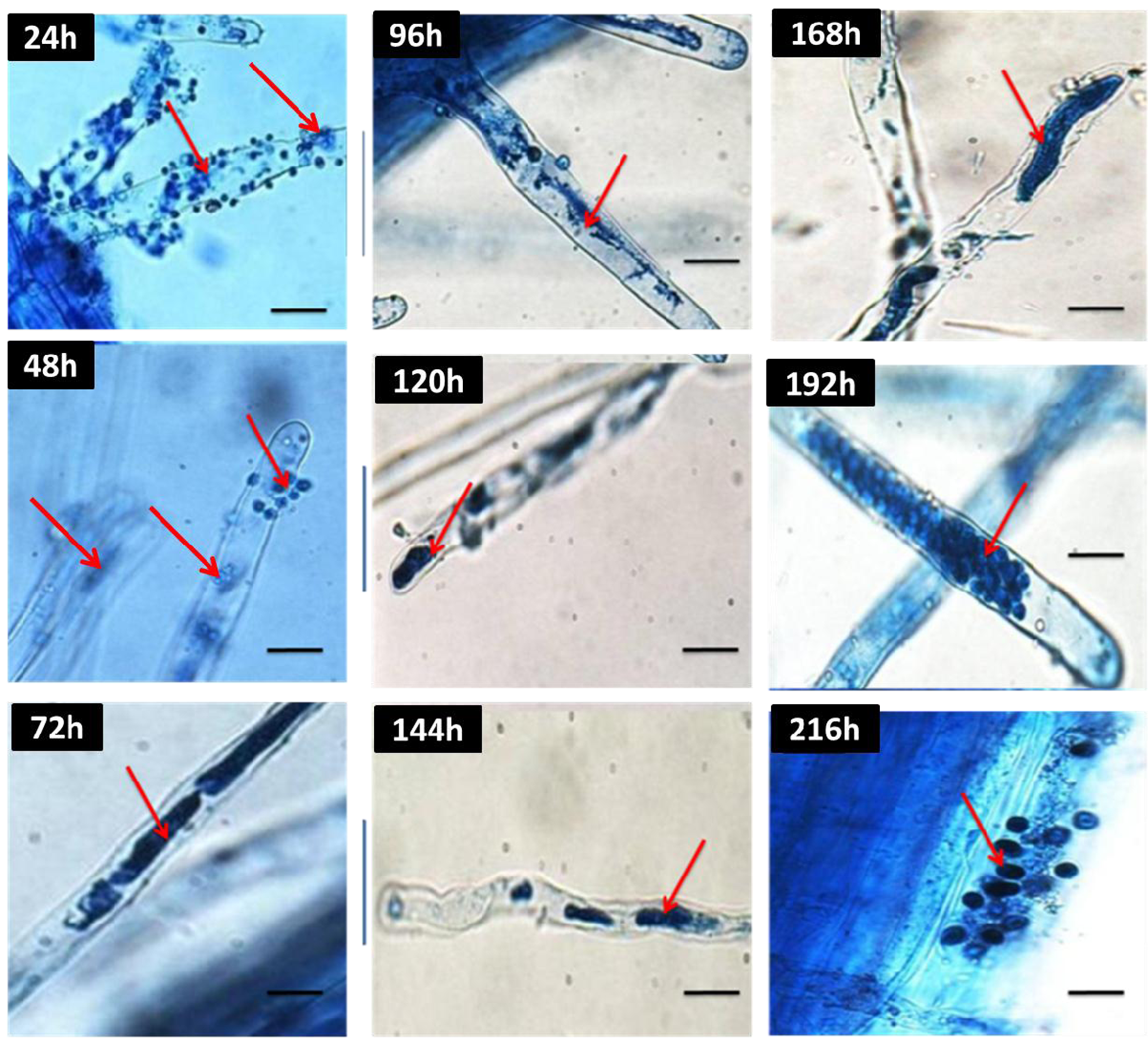

Supplement: Figure S1 — The early process of A. thaliana infection by P. brassicae stained with trypan blue. The red arrows point to P. brassicae at different stages (1–2d, primary zoospore; 3–7d, primary plasmodial; 8d, zoosporangia; 9d, secondary zoospores; and 10–15d, secondary plasmodia). [file Image1.TIF]

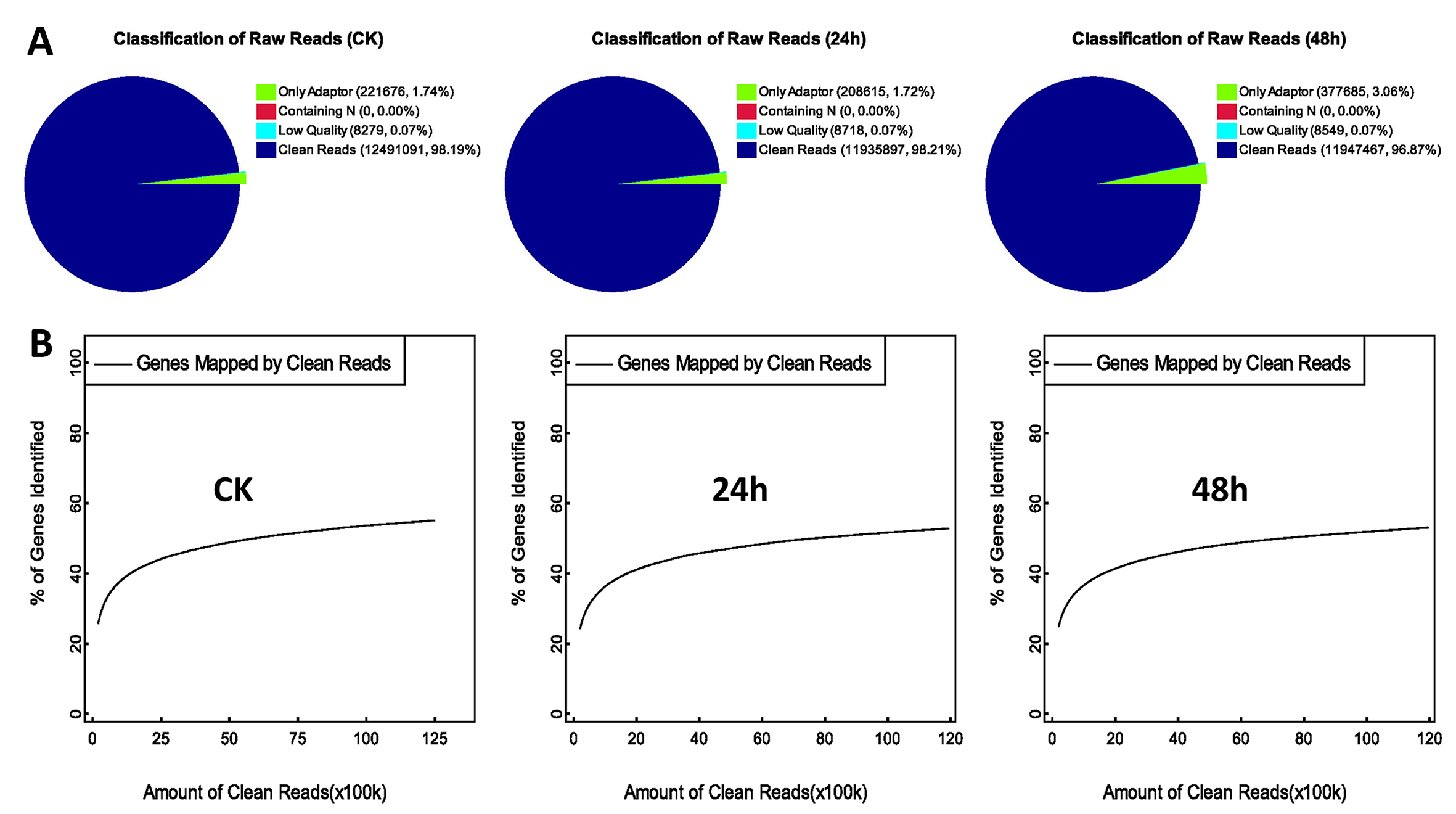

Supplement: Figure S2 — Evaluation of sequencing quality (A) and saturation (B) analysis of DEGs in A. thaliana during early infection by P. brassicae. [file Image2.TIF]

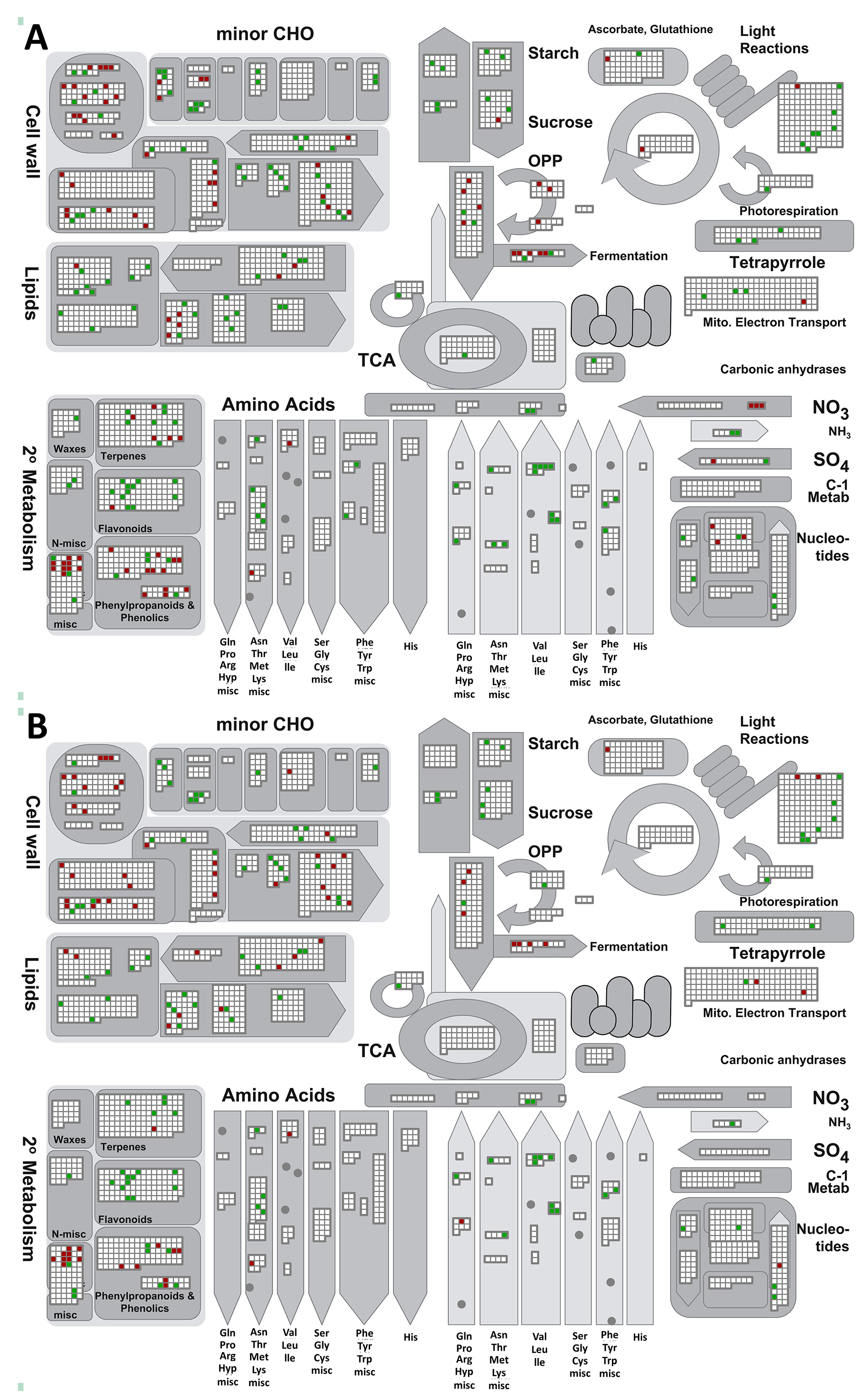

Supplement: Figure S3 — Metabolism pathway analyses of DEGs in A. thaliana during early infection by P. brassicae. Metabolism pathway analysis of the DEGs was performed using MAPMAN software. Red boxes mean up-regulated genes and green mean down-regulated genes. (A) 24 h after inoculation; (B) 48 h after inoculation. The pathway frames are from MAPMAN software database. [file Image3.TIF]

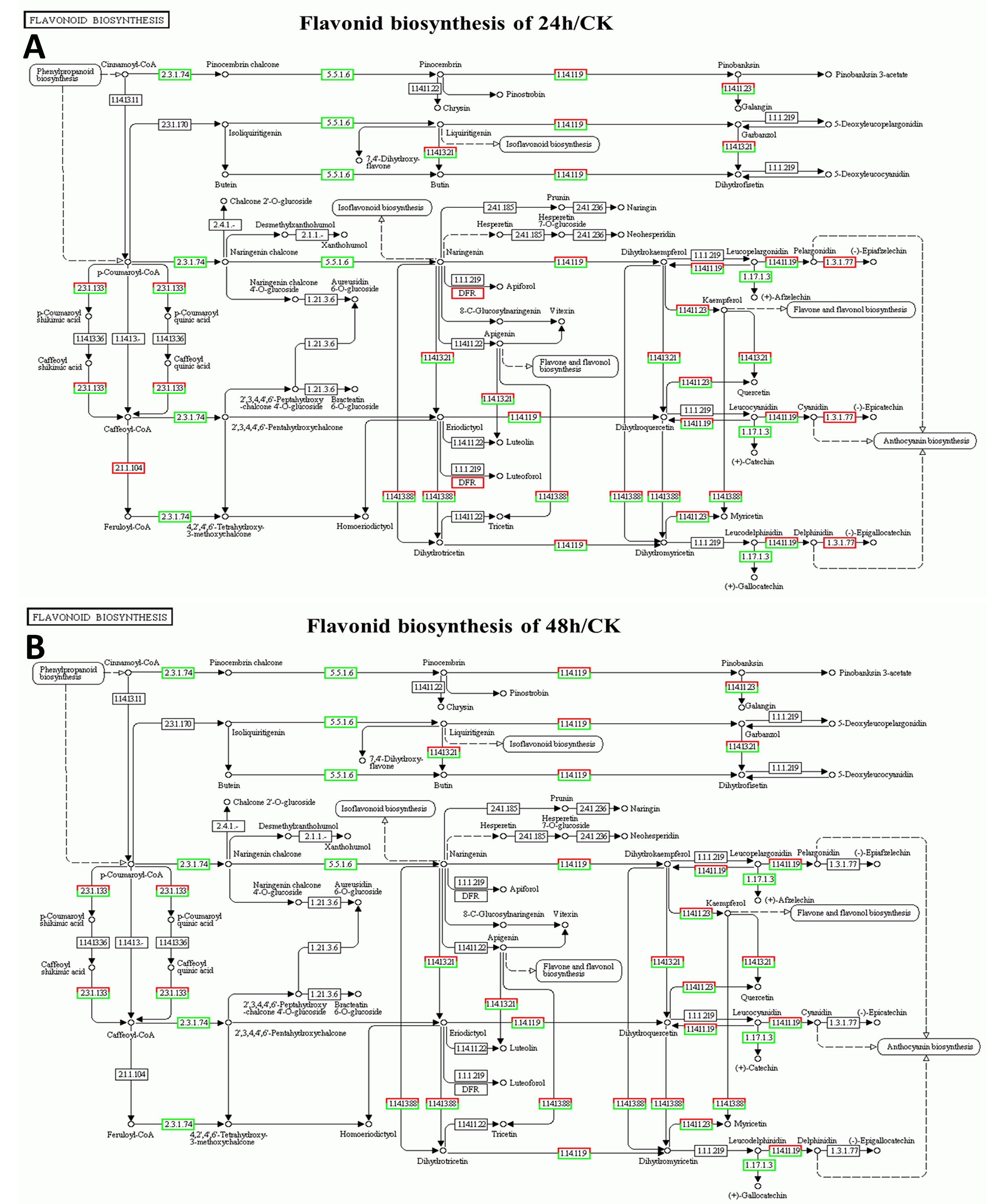

Supplement: Figure S4 — Flavonoid biosynthesis pathway analyses of DEGs in A. thaliana during early infection by P. brassicae. Analysis of the flavonoid biosynthesis pathway of DEGs was performed. Red boxes mean up-regulated genes and green mean down-regulated genes. (A) 24 h after inoculation; (B) 48 h after inoculation. [file Image4.TIF]

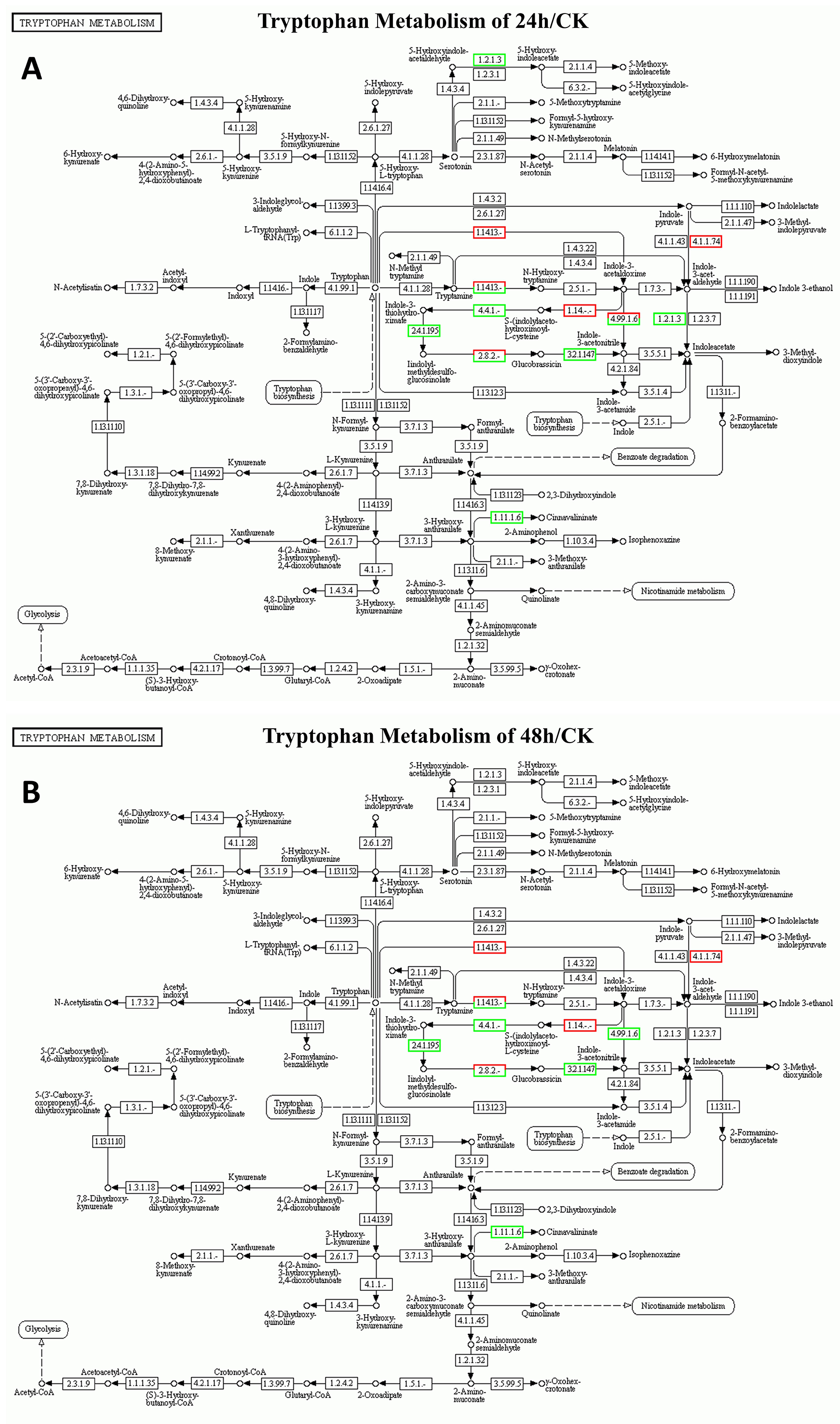

Supplement: Figure S5 — Tryptophan metabolism pathway analyses of DEGs in A. thaliana during early infection by P. brassicae. Analysis of the tryptophan metabolism pathway of DEGs was performed. Red boxes mean up-regulated genes and green mean down-regulated genes. (A) 24 h after inoculation; (B) 48 h after inoculation. [file Image5.TIF]

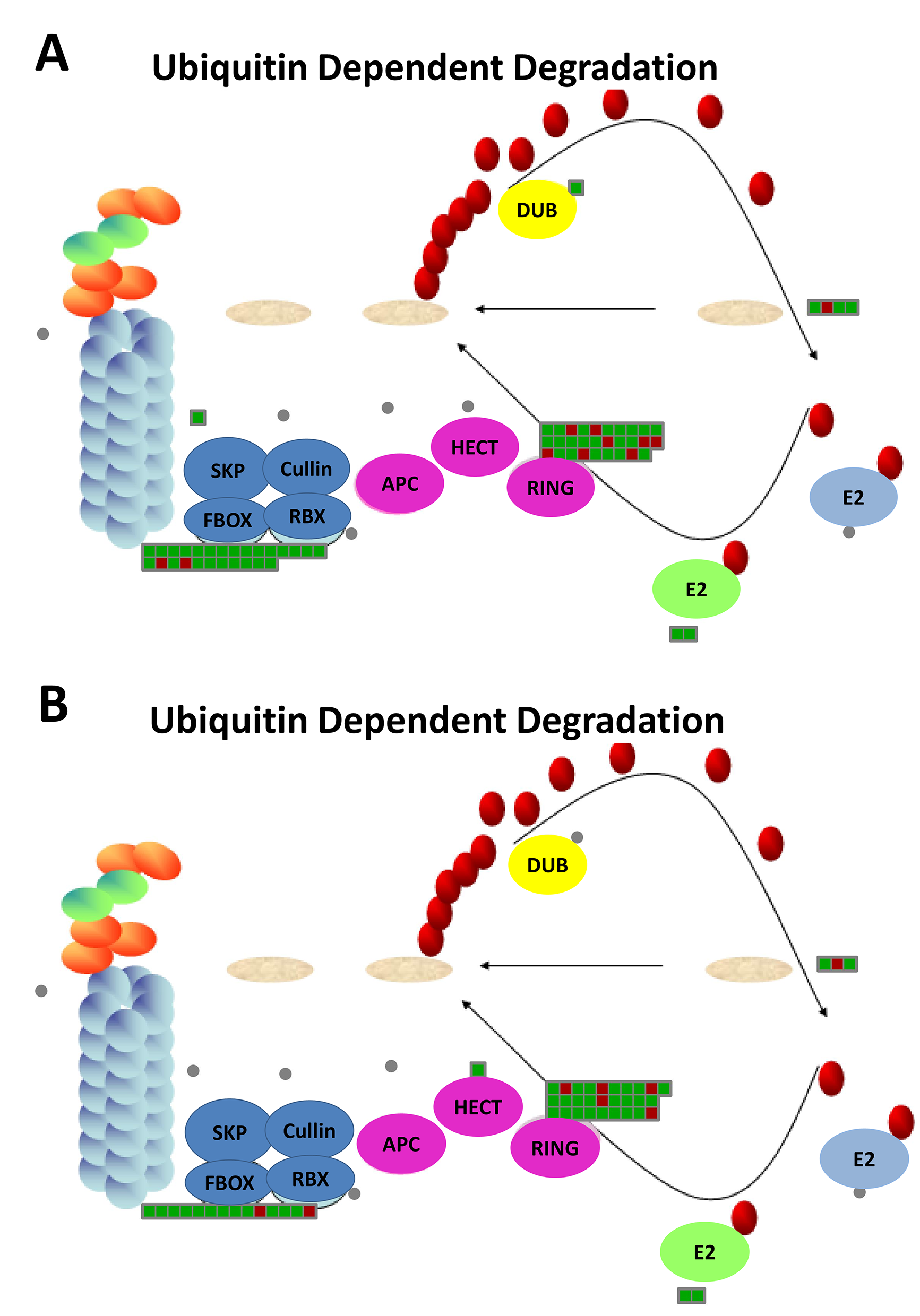

Supplement: Figure S6 — Ubiquitin pathway analyses of DEGs in A. thaliana during early infection by P. brassicae. Analysis of the ubiquitin pathway of DEGs was performed using MAPMAN software. Red boxes mean up-regulated genes and green mean down-regulated genes. (A) 24 h after inoculation; (B) 48 h after inoculation. The pathway frames are from the MAPMAN software database. [file Image6.TIF]

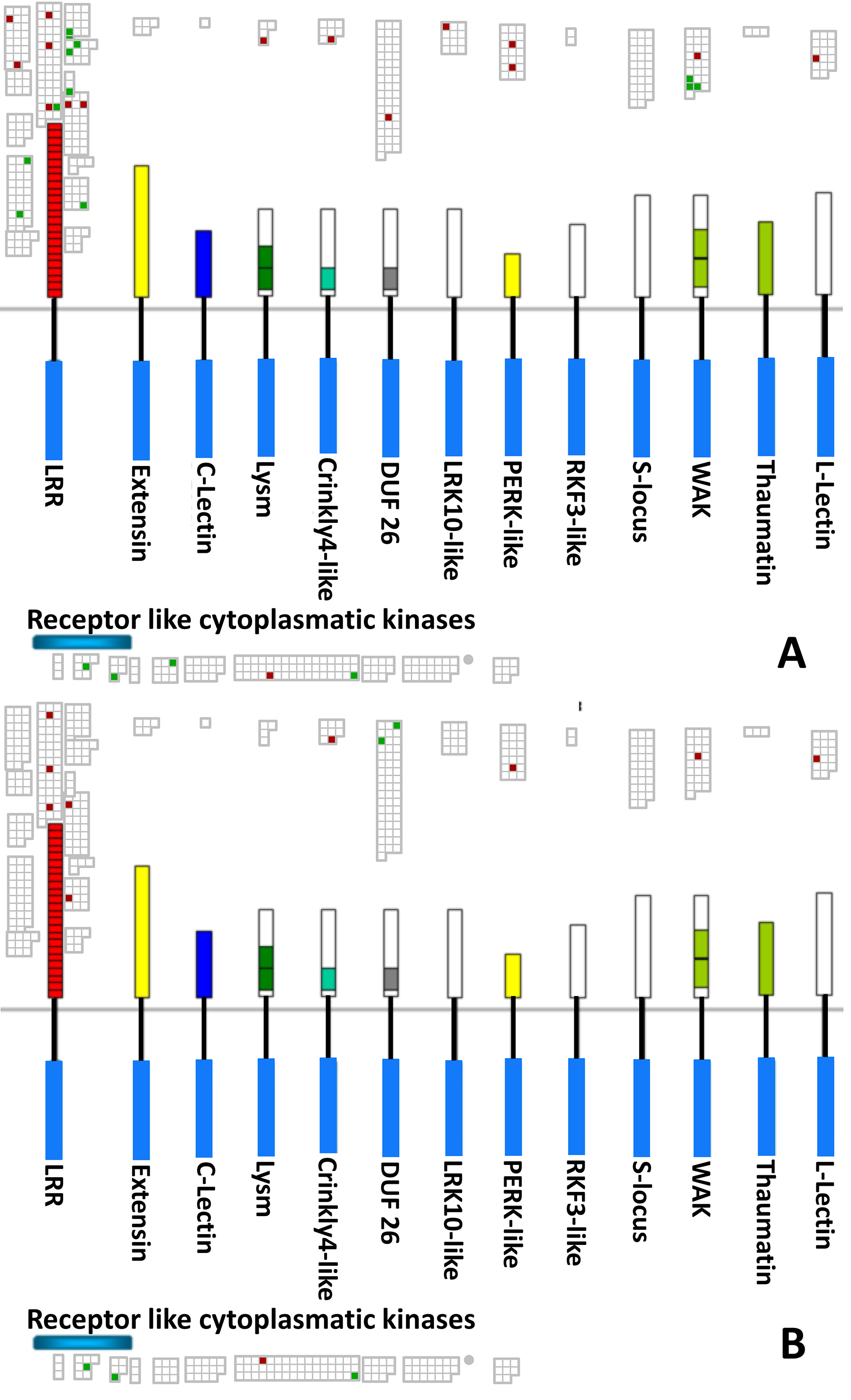

Supplement: Figure S7 — Receptor-like kinases gene analyses of DEGs in A. thaliana during early infection by P. brassicae. Analysis of the receptor-like kinases pathways of DEGs were performed using MAPMAN software. Red boxes mean up-regulated genes and green mean down-regulated genes. (A) 24 h after inoculation; (B) 48 h after inoculation. The pathway frames are from the MAPMAN software database. [file Image7.TIF]
