# Supplementary material for: Transcriptome Analysis of Arabidopsis thaliana in Response to Plasmodiophora brassicae during Early Infection
Source: Front Microbiol. 2017 Apr 24;8:673. doi: 10.3389/fmicb.2017.00673 (PMC5401899; doi:10.3389/fmicb.2017.00673)
Supplement: Table S1 — The genes and primers used in qRT-PCR. [file DataSheet1.DOCX]

**Supplementary Table 1 Primers used in this study**

| **Gene No.** | **Sense Primer** | **Anti-sense Primer** | **Gene class** |
| --- | --- | --- | --- |
| AT1G50060 | GCCTACGCATTGAACTATT | AATCTCTCCACACCACTT | PR Protein |
| AT1G69010 | CCAATCAAGGTCTAACTCAT | ACACGATACAAGGAATCTG | PR Protein |
| AT1G73620 | GTCTACAAGTCCGTTCAC | GCGTATGAGTAAGCCTTAG | PR Protein |
| AT2G19970 | CCGTCCTTATTGATTATATTGATG | CACAGCAGCAACTTATACA | PR Protein |
| AT2G19990 | ATGTCAAACCGCAAGAAA | TCCGTCATCCAATACTCA | PR Protein |
| AT2G22770 | TTGTTGATTAGAGTCCATTGT | TTATGTTCTTCACCACTTCTT | PR Protein |
| AT2G40200 | AACAATCCAAGCAGAGATAA | AGAAGTAGTCGTAGAAGAAGA | PR Protein |
| AT3G04720 | GGTGAAGAACACAAGAACA | TGGTAGTCAACAATGAGATG | PR Protein |
| AT3G12500 | ATGGAGTGATTACGAACATC | TCAAGATTACCACCAGGAT | PR Protein |
| AT4G33710 | TGGTCATTATACTCAAGTTGT | GTCTGCGTACACATATAGTTA | PR Protein |
| AT5G52260 | AATCCAACTTCATCACCATC | TCATCATCACTTCCTCTTCA | MYB |
| AT1G71030 | CAATGGTTCAACAGGTCAA | CGGTTATCGTCGGTTAGT | MYB |
| AT5G37260 | CTGAAGATGGCAAGAAGAAG | TTGAACTCCGACACTACC | MYB |
| AT4G01250 | GACCACTATTGCTACTTATTCAT | ATCGCTAACCACCGTATC | WRKY |
| AT1G16400 | GGACAATTATGATGGCTATGAT | CAAGTAATACAGAAACGACAAAG | IAA |
| AT1G22400 | AGATAGGTGGAGATGTGAAG | CATAACGGAAGAACCAAGTT | IAA |
| AT1G62570 | ATATGAGAGTTGATGACAATCG | GGAATACCTAACGCTTCTTG | IAA |
| AT1G77380 | TTATCAAGAACAGTCAAGAGAAC | GATTATAGAGAAGAACTAACAGCAT | IAA |
| AT4G13770 | TCCTTACTTCAGAGCCTTAG | AGTCAACTTCCTTCTCAAGA | IAA |
| AT4G33070 | GAGATACGAAGAGGAGTTAGT | ATTGGTAAGAAGCATTGAAGAT | IAA |
| AT5G07010 | TTAGTGGATGACAAGTTAGGT | AACAACAATACAACTGCCTTA | IAA |
| AT1G55020 | TTGAAGAACTTGAGAAGAATCC | TCGTCGCTAGAATGAGTAG | JA |
| AT1G68040 | ATACCTTCAACATACCTATCTACT | ACCATCCAATGCCTAACA | SA |
| AT1G22400 | AGATAGGTGGAGATGTGAAG | CATAACGGAAGAACCAAGTT | cytokinin |
| AT3G23630 | GATTCAACGGCTTCACAA | GCTCTACTTCCACTTCCT | cytokinin |
| AT3G50740 | GAAGGTGCTGAGATGAGA | GCGATTCTTGACAACGATT | phenylpropanoids |
| AT2G30210 | GCATTGTCACATTGATTCAC | AATAGAGCAGTTGTCCATCT | simple phenols |
| AT5G07130 | AATACAGACACATCCTCCTT | CCTTCTCCGTTCTCTACTAA | simple phenols |
| AT4G23590 | CAACTCTCACCTGCTACA | AATGCCTCAACCAGTTATTC | isoprenoids |
| AT5G23010 | CAAGCACATAAGCCCATAG | GAGAAGACAGCGTTCAATT | glucosinolates |
| AT2G43100 | GCTTAATCCTCTTCCATAACC | ACTCACCATATTCACATATCTTG | glucosinolates |
| AT3G58990 | CACAAGGAAGACGGTAGTA | GATTACATTCAAGCAGAAGGA | glucosinolates |
| AT1G16400 | AAGGAGACGGAATCACAA | CCAACATCATAGCCATCATAA | glucosinolates |
| AT4G13770 | TCTTGAGAAGGAAGTTGACT | GAGGAGAAGGTTCGCATA | glucosinolates |
| AT1G62560 | AGACATACTCATACGATAGGTAG | TCAACACTAATCAACTTCTTCC | glucosinolates |
| AT1G62570 | TGGAAGAAGCATACGAAGA | CCTAGCCAAGAAGACCTT | glucosinolates |
| AT4G26220 | GTGGAGGATAGAAGTCAAGA | ACAATACTCGCTCAATATAACC | Cell Wall |
| AT4G36220 | GCCTTAACGGAGTTATTACG | CGATGTCGGATTCTTCAAC | Cell Wall |
| AT4G37970 | GCAAGAGACACAAGAGATG | GAGTAGCAGCCAATGTATTAG | Cell Wall |
| AT4G39330 | ATTGGTGGTGATGATGTCTA | ACTGATGGAATGCTGGAA | Cell Wall |
| AT3G18780  (Actin) | AATCCAACTTCATCACCATC | TCATCATCACTTCCTCTTCA | Actin |

**Supplementary Table 2 The statistical results mapped to genes of sample CK, 24h and 48h**

**CK:**

| **Map to Gene** | **Reads number** | **Percentage** |
| --- | --- | --- |
| Total Reads | 12491091 | 100.00% |
| Total Base Pairs | 612063459 | 100.00% |
| Total Mapped Reads | 11750320 | **94.07%** |
| perfect match | 10612536 | **84.96%** |
| <=2bp mismatch | 1137784 | 9.11% |
| unique match | 7541026 | 60.37% |
| multi-position match | 4209294 | 33.70% |
| Total Unmapped Reads | 740771 | **5.93%** |

**24h:**

| **Map to Gene** | **Reads number** | **Percentage** |
| --- | --- | --- |
| Total Reads | 11935897 | 100.00% |
| Total Base Pairs | 584858953 | 100.00% |
| Total Mapped Reads | 11154593 | **93.45%** |
| perfect match | 10073510 | **84.40%** |
| <=2bp mismatch | 1081083 | 9.06% |
| unique match | 7393626 | 61.94% |
| multi-position match | 3760967 | 31.51% |
| Total Unmapped Reads | 781304 | **6.55%** |

**48h:**

| **Map to Gene** | **Reads number** | **Percentage** |
| --- | --- | --- |
| Total Reads | 11947467 | 100.00% |
| Total Base Pairs | 585425883 | 100.00% |
| Total Mapped Reads | 11184974 | **93.62%** |
| perfect match | 10040366 | **84.04%** |
| <=2bp mismatch | 1144608 | 9.58% |
| unique match | 7315361 | 61.23% |
| multi-position match | 3869613 | 32.39% |
| Total Unmapped Reads | 762493 | **6.38%** |

**Supplementary Table 3 The significantly enriched GO terms of DEGs (P≤0.05)**

**Molecular Function (24h VS CK):**

| **Gene Ontology term** | **Cluster frequency** | **Genome frequency of use** | **Corrected P-value** |
| --- | --- | --- | --- |
| [Oxidoreductase activity](http://amigo.geneontology.org/cgi-bin/amigo/go.cgi?action=query&view=query&query=GO:0016491&search_constraint=terms) | 153 out of 1095 genes, 14.0% | 2087 out of 20303 genes, 10.3% | 0.01197 |
| [Arsenate reductase activity](http://amigo.geneontology.org/cgi-bin/amigo/go.cgi?action=query&view=query&query=GO:0030611&search_constraint=terms) | 5 out of 1095 genes, 0.5% | 9 out of 20303 genes, 0.0% | 0.01296 |
| [Oxidoreductase activity, acting on single donors with incorporation of molecular oxygen](http://amigo.geneontology.org/cgi-bin/amigo/go.cgi?action=query&view=query&query=GO:0016701&search_constraint=terms) | 15 out of 1095 genes, 1.4% | 91 out of 20303 genes, 0.4% | 0.02730 |

**Molecular Function (48h VS CK):**

| **Gene Ontology term** | **Cluster frequency** | **Genome frequency of use** | **Corrected P-value** |
| --- | --- | --- | --- |
| [Oxidoreductase activity](http://amigo.geneontology.org/cgi-bin/amigo/go.cgi?action=query&view=query&query=GO:0016491&search_constraint=terms) | 119 out of 771 genes, 15.4% | 2087 out of 20303 genes, 10.3% | 0.00081 |
| [Arsenate reductase activity](http://amigo.geneontology.org/cgi-bin/amigo/go.cgi?action=query&view=query&query=GO:0030611&search_constraint=terms) | 5 out of 771 genes, 0.6% | 9 out of 20303 genes, 0.0% | 0.00192 |
| [Transferase activity, transferring hexosyl groups](http://amigo.geneontology.org/cgi-bin/amigo/go.cgi?action=query&view=query&query=GO:0016758&search_constraint=terms) | 32 out of 771 genes, 4.2% | 391 out of 20303 genes, 1.9% | 0.00927 |
| [UDP-glucosyltransferase activity](http://amigo.geneontology.org/cgi-bin/amigo/go.cgi?action=query&view=query&query=GO:0035251&search_constraint=terms) | 18 out of 771 genes, 2.3% | 168 out of 20303 genes, 0.8% | 0.01636 |
| [Glucosyltransferase activity](http://amigo.geneontology.org/cgi-bin/amigo/go.cgi?action=query&view=query&query=GO:0046527&search_constraint=terms) | 21 out of 771 genes, 2.7% | 217 out of 20303 genes, 1.1% | 0.01903 |

**Biological Processes (24h VS CK):**

| **Gene Ontology term** | **Cluster frequency** | **Genome frequency of use** | **Corrected P-value** |
| --- | --- | --- | --- |
| [Response to stimulus](http://amigo.geneontology.org/cgi-bin/amigo/go.cgi?action=query&view=query&query=GO:0050896&search_constraint=terms) | 497 out of 1054 genes, 47.2% | 6257 out of 18568 genes, 33.7% | 6.44e-18 |
| [Response to chemical stimulus](http://amigo.geneontology.org/cgi-bin/amigo/go.cgi?action=query&view=query&query=GO:0042221&search_constraint=terms) | 263 out of 1054 genes, 25.0% | 3108 out of 18568 genes, 16.7% | 1.19e-09 |
| [Response to stress](http://amigo.geneontology.org/cgi-bin/amigo/go.cgi?action=query&view=query&query=GO:0006950&search_constraint=terms) | 288 out of 1054 genes, 27.3% | 3533 out of 18568 genes, 19.0% | 5.76e-09 |
| [Response to organic substance](http://amigo.geneontology.org/cgi-bin/amigo/go.cgi?action=query&view=query&query=GO:0010033&search_constraint=terms) | 179 out of 1054 genes, 17.0% | 1968 out of 18568 genes, 10.6% | 4.09e-08 |
| [Response to abiotic stimulus](http://amigo.geneontology.org/cgi-bin/amigo/go.cgi?action=query&view=query&query=GO:0009628&search_constraint=terms) | 171 out of 1054 genes, 16.2% | 1981 out of 18568 genes, 10.7% | 6.23e-06 |
| [Response to endogenous stimulus](http://amigo.geneontology.org/cgi-bin/amigo/go.cgi?action=query&view=query&query=GO:0009719&search_constraint=terms) | 147 out of 1054 genes, 13.9% | 1658 out of 18568 genes, 8.9% | 1.55e-05 |
| [Response to hormone stimulus](http://amigo.geneontology.org/cgi-bin/amigo/go.cgi?action=query&view=query&query=GO:0009725&search_constraint=terms) | 133 out of 1054 genes, 12.6% | 1505 out of 18568 genes, 8.1% | 9.65e-05 |
| [Response to oxidative stress](http://amigo.geneontology.org/cgi-bin/amigo/go.cgi?action=query&view=query&query=GO:0006979&search_constraint=terms) | 24 out of 1054 genes, 2.3% | 126 out of 18568 genes, 0.7% | 9.93e-05 |
| [Response to reactive oxygen species](http://amigo.geneontology.org/cgi-bin/amigo/go.cgi?action=query&view=query&query=GO:0000302&search_constraint=terms) | 20 out of 1054 genes, 1.9% | 93 out of 18568 genes, 0.5% | 0.00013 |
| [Response to carbohydrate stimulus](http://amigo.geneontology.org/cgi-bin/amigo/go.cgi?action=query&view=query&query=GO:0009743&search_constraint=terms) | 52 out of 1054 genes, 4.9% | 443 out of 18568 genes, 2.4% | 0.00036 |
| [Secondary metabolic process](http://amigo.geneontology.org/cgi-bin/amigo/go.cgi?action=query&view=query&query=GO:0019748&search_constraint=terms) | 72 out of 1054 genes, 6.8% | 708 out of 18568 genes, 3.8% | 0.00071 |
| [Response to light intensity](http://amigo.geneontology.org/cgi-bin/amigo/go.cgi?action=query&view=query&query=GO:0009642&search_constraint=terms) | 21 out of 1054 genes, 2.0% | 124 out of 18568 genes, 0.7% | 0.00419 |
| [Response to light stimulus](http://amigo.geneontology.org/cgi-bin/amigo/go.cgi?action=query&view=query&query=GO:0009416&search_constraint=terms) | 69 out of 1054 genes, 6.5% | 726 out of 18568 genes, 3.9% | 0.01202 |
| [Defense response](http://amigo.geneontology.org/cgi-bin/amigo/go.cgi?action=query&view=query&query=GO:0006952&search_constraint=terms) | 85 out of 1054 genes, 8.1% | 976 out of 18568 genes, 5.3% | 0.03409 |
| [Response to radiation](http://amigo.geneontology.org/cgi-bin/amigo/go.cgi?action=query&view=query&query=GO:0009314&search_constraint=terms) | 82 out of 1054 genes, 7.8% | 935 out of 18568 genes, 5.0% | 0.03650 |

**Biological Processes (48h VS CK):**

| **Gene Ontology term** | **Cluster frequency** | **Genome frequency of use** | **Corrected P-value** |
| --- | --- | --- | --- |
| [Response to stimulus](http://amigo.geneontology.org/cgi-bin/amigo/go.cgi?action=query&view=query&query=GO:0050896&search_constraint=terms) | 362 out of 767 genes, 47.2% | 6257 out of 18568 genes, 33.7% | 1.11e-12 |
| [Response to organic substance](http://amigo.geneontology.org/cgi-bin/amigo/go.cgi?action=query&view=query&query=GO:0010033&search_constraint=terms) | 140 out of 767 genes, 18.3% | 1968 out of 18568 genes, 10.6% | 3.19e-08 |
| [Response to endogenous stimulus](http://amigo.geneontology.org/cgi-bin/amigo/go.cgi?action=query&view=query&query=GO:0009719&search_constraint=terms) | 123 out of 767 genes, 16.0% | 1658 out of 18568 genes, 8.9% | 4.31e-08 |
| [Response to chemical stimulus](http://amigo.geneontology.org/cgi-bin/amigo/go.cgi?action=query&view=query&query=GO:0042221&search_constraint=terms) | 197 out of 767 genes, 25.7% | 3108 out of 18568 genes, 16.7% | 5.19e-08 |
| [Response to stress](http://amigo.geneontology.org/cgi-bin/amigo/go.cgi?action=query&view=query&query=GO:0006950&search_constraint=terms) | 214 out of 767 genes, 27.9% | 3533 out of 18568 genes, 19.0% | 3.72e-07 |
| [Response to hormone stimulus](http://amigo.geneontology.org/cgi-bin/amigo/go.cgi?action=query&view=query&query=GO:0009725&search_constraint=terms) | 110 out of 767 genes, 14.3% | 1505 out of 18568 genes, 8.1% | 1.20e-06 |
| [Secondary metabolic process](http://amigo.geneontology.org/cgi-bin/amigo/go.cgi?action=query&view=query&query=GO:0019748&search_constraint=terms) | 61 out of 767 genes, 8.0% | 708 out of 18568 genes, 3.8% | 2.49e-05 |
| [Response to abiotic stimulus](http://amigo.geneontology.org/cgi-bin/amigo/go.cgi?action=query&view=query&query=GO:0009628&search_constraint=terms) | 130 out of 767 genes, 16.9% | 1981 out of 18568 genes, 10.7% | 2.66e-05 |
| [Response to osmotic stress](http://amigo.geneontology.org/cgi-bin/amigo/go.cgi?action=query&view=query&query=GO:0006970&search_constraint=terms) | 69 out of 767 genes, 9.0% | 881 out of 18568 genes, 4.7% | 0.00012 |
| [Peptide transport](http://amigo.geneontology.org/cgi-bin/amigo/go.cgi?action=query&view=query&query=GO:0015833&search_constraint=terms) | 12 out of 767 genes, 1.6% | 61 out of 18568 genes, 0.3% | 0.00336 |
| [Response to red or far red light](http://amigo.geneontology.org/cgi-bin/amigo/go.cgi?action=query&view=query&query=GO:0009639&search_constraint=terms) | 28 out of 767 genes, 3.7% | 277 out of 18568 genes, 1.5% | 0.00681 |
| [Phenylpropanoid metabolic process](http://amigo.geneontology.org/cgi-bin/amigo/go.cgi?action=query&view=query&query=GO:0009698&search_constraint=terms) | 29 out of 767 genes, 3.8% | 296 out of 18568 genes, 1.6% | 0.00870 |
| [Response to carbohydrate stimulus](http://amigo.geneontology.org/cgi-bin/amigo/go.cgi?action=query&view=query&query=GO:0009743&search_constraint=terms) | 37 out of 767 genes, 4.8% | 443 out of 18568 genes, 2.4% | 0.02268 |
| [Response to light stimulus](http://amigo.geneontology.org/cgi-bin/amigo/go.cgi?action=query&view=query&query=GO:0009416&search_constraint=terms) | 52 out of 767 genes, 6.8% | 726 out of 18568 genes, 3.9% | 0.04542 |
| [External encapsulating structure organization](http://amigo.geneontology.org/cgi-bin/amigo/go.cgi?action=query&view=query&query=GO:0045229&search_constraint=terms) | 18 out of 767 genes, 2.3% | 157 out of 18568 genes, 0.8% | 0.04825 |

**Supplementary Table 4 The significantly enriched pathways of DEGs (P≤0.05)**

**Pathway (24h VS CK):**

| **#** | **Pathway** | **DEGs with pathway annotation (921)** | **All genes with pathway annotation (18086)** | **P value** | **Q value** | **Pathway ID** |
| --- | --- | --- | --- | --- | --- | --- |
| 1 | [Flavonoid biosynthesis](file:///E:\%E8%AF%95%E9%AA%8C%E7%BB%93%E6%9E%9C%E6%95%B0%E6%8D%AE\%E6%A0%B9%E8%82%BF%E4%BE%B5%E6%9F%93%E6%8B%9F%E5%8D%97%E8%8A%A5\%E6%8B%9F%E5%8D%97%E8%8A%A5%E5%AD%97%E8%A1%A8%E8%BE%BE%E8%B0%B1%E7%BB%93%E6%9E%9C%E6%95%B0%E6%8D%AE\%E8%A1%A8%E8%BE%BE%E8%B0%B1%E7%BB%93%E6%9E%9C\Arabidopsis_thaliana\GeneDiffExp\Pathway\CK-VS-24h.htm#gene1) | 33 (3.58)% | 302 (1.67)% | 3.100173e-05 | 0.002433290 | ko00941 |
| 2 | [Tryptophan metabolism](file:///E:\%E8%AF%95%E9%AA%8C%E7%BB%93%E6%9E%9C%E6%95%B0%E6%8D%AE\%E6%A0%B9%E8%82%BF%E4%BE%B5%E6%9F%93%E6%8B%9F%E5%8D%97%E8%8A%A5\%E6%8B%9F%E5%8D%97%E8%8A%A5%E5%AD%97%E8%A1%A8%E8%BE%BE%E8%B0%B1%E7%BB%93%E6%9E%9C%E6%95%B0%E6%8D%AE\%E8%A1%A8%E8%BE%BE%E8%B0%B1%E7%BB%93%E6%9E%9C\Arabidopsis_thaliana\GeneDiffExp\Pathway\CK-VS-24h.htm#gene2) | 25 (2.71)% | 208 (1.15)% | 5.977545e-05 | 0.002433290 | ko00380 |
| 3 | [Biosynthesis of secondary metabolites](file:///E:\%E8%AF%95%E9%AA%8C%E7%BB%93%E6%9E%9C%E6%95%B0%E6%8D%AE\%E6%A0%B9%E8%82%BF%E4%BE%B5%E6%9F%93%E6%8B%9F%E5%8D%97%E8%8A%A5\%E6%8B%9F%E5%8D%97%E8%8A%A5%E5%AD%97%E8%A1%A8%E8%BE%BE%E8%B0%B1%E7%BB%93%E6%9E%9C%E6%95%B0%E6%8D%AE\%E8%A1%A8%E8%BE%BE%E8%B0%B1%E7%BB%93%E6%9E%9C\Arabidopsis_thaliana\GeneDiffExp\Pathway\CK-VS-24h.htm#gene3) | 166 (18.02)% | 2469 (13.65)% | 7.735566e-05 | 0.002433290 | ko01110 |
| 4 | [Circadian rhythm - plant](file:///E:\%E8%AF%95%E9%AA%8C%E7%BB%93%E6%9E%9C%E6%95%B0%E6%8D%AE\%E6%A0%B9%E8%82%BF%E4%BE%B5%E6%9F%93%E6%8B%9F%E5%8D%97%E8%8A%A5\%E6%8B%9F%E5%8D%97%E8%8A%A5%E5%AD%97%E8%A1%A8%E8%BE%BE%E8%B0%B1%E7%BB%93%E6%9E%9C%E6%95%B0%E6%8D%AE\%E8%A1%A8%E8%BE%BE%E8%B0%B1%E7%BB%93%E6%9E%9C\Arabidopsis_thaliana\GeneDiffExp\Pathway\CK-VS-24h.htm#gene4) | 34 (3.69)% | 332 (1.84)% | 8.768611e-05 | 0.002433290 | ko04712 |
| 5 | [Starch and sucrose metabolism](file:///E:\%E8%AF%95%E9%AA%8C%E7%BB%93%E6%9E%9C%E6%95%B0%E6%8D%AE\%E6%A0%B9%E8%82%BF%E4%BE%B5%E6%9F%93%E6%8B%9F%E5%8D%97%E8%8A%A5\%E6%8B%9F%E5%8D%97%E8%8A%A5%E5%AD%97%E8%A1%A8%E8%BE%BE%E8%B0%B1%E7%BB%93%E6%9E%9C%E6%95%B0%E6%8D%AE\%E8%A1%A8%E8%BE%BE%E8%B0%B1%E7%BB%93%E6%9E%9C\Arabidopsis_thaliana\GeneDiffExp\Pathway\CK-VS-24h.htm#gene5) | 43 (4.67)% | 482 (2.67)% | 0.0002612799 | 0.005800414 | ko00500 |
| 6 | [Stilbenoid, diarylheptanoid and gingerol biosynthesis](file:///E:\%E8%AF%95%E9%AA%8C%E7%BB%93%E6%9E%9C%E6%95%B0%E6%8D%AE\%E6%A0%B9%E8%82%BF%E4%BE%B5%E6%9F%93%E6%8B%9F%E5%8D%97%E8%8A%A5\%E6%8B%9F%E5%8D%97%E8%8A%A5%E5%AD%97%E8%A1%A8%E8%BE%BE%E8%B0%B1%E7%BB%93%E6%9E%9C%E6%95%B0%E6%8D%AE\%E8%A1%A8%E8%BE%BE%E8%B0%B1%E7%BB%93%E6%9E%9C\Arabidopsis_thaliana\GeneDiffExp\Pathway\CK-VS-24h.htm#gene6) | 30 (3.26)% | 308 (1.7)% | 0.0005232152 | 0.009679481 | ko00945 |
| 7 | [Glucosinolate biosynthesis](file:///E:\%E8%AF%95%E9%AA%8C%E7%BB%93%E6%9E%9C%E6%95%B0%E6%8D%AE\%E6%A0%B9%E8%82%BF%E4%BE%B5%E6%9F%93%E6%8B%9F%E5%8D%97%E8%8A%A5\%E6%8B%9F%E5%8D%97%E8%8A%A5%E5%AD%97%E8%A1%A8%E8%BE%BE%E8%B0%B1%E7%BB%93%E6%9E%9C%E6%95%B0%E6%8D%AE\%E8%A1%A8%E8%BE%BE%E8%B0%B1%E7%BB%93%E6%9E%9C\Arabidopsis_thaliana\GeneDiffExp\Pathway\CK-VS-24h.htm#gene7) | 13 (1.41)% | 92 (0.51)% | 0.000759441 | 0.012042564 | ko00966 |
| 8 | [Alanine, aspartate and glutamate metabolism](file:///E:\%E8%AF%95%E9%AA%8C%E7%BB%93%E6%9E%9C%E6%95%B0%E6%8D%AE\%E6%A0%B9%E8%82%BF%E4%BE%B5%E6%9F%93%E6%8B%9F%E5%8D%97%E8%8A%A5\%E6%8B%9F%E5%8D%97%E8%8A%A5%E5%AD%97%E8%A1%A8%E8%BE%BE%E8%B0%B1%E7%BB%93%E6%9E%9C%E6%95%B0%E6%8D%AE\%E8%A1%A8%E8%BE%BE%E8%B0%B1%E7%BB%93%E6%9E%9C\Arabidopsis_thaliana\GeneDiffExp\Pathway\CK-VS-24h.htm#gene8) | 16 (1.74)% | 141 (0.78)% | 0.002217184 | 0.030763428 | ko00250 |
| 9 | [Diterpenoid biosynthesis](file:///E:\%E8%AF%95%E9%AA%8C%E7%BB%93%E6%9E%9C%E6%95%B0%E6%8D%AE\%E6%A0%B9%E8%82%BF%E4%BE%B5%E6%9F%93%E6%8B%9F%E5%8D%97%E8%8A%A5\%E6%8B%9F%E5%8D%97%E8%8A%A5%E5%AD%97%E8%A1%A8%E8%BE%BE%E8%B0%B1%E7%BB%93%E6%9E%9C%E6%95%B0%E6%8D%AE\%E8%A1%A8%E8%BE%BE%E8%B0%B1%E7%BB%93%E6%9E%9C\Arabidopsis_thaliana\GeneDiffExp\Pathway\CK-VS-24h.htm#gene9) | 12 (1.3)% | 95 (0.53)% | 0.003152958 | 0.037832947 | ko00904 |
| 10 | [Phenylpropanoid biosynthesis](file:///E:\%E8%AF%95%E9%AA%8C%E7%BB%93%E6%9E%9C%E6%95%B0%E6%8D%AE\%E6%A0%B9%E8%82%BF%E4%BE%B5%E6%9F%93%E6%8B%9F%E5%8D%97%E8%8A%A5\%E6%8B%9F%E5%8D%97%E8%8A%A5%E5%AD%97%E8%A1%A8%E8%BE%BE%E8%B0%B1%E7%BB%93%E6%9E%9C%E6%95%B0%E6%8D%AE\%E8%A1%A8%E8%BE%BE%E8%B0%B1%E7%BB%93%E6%9E%9C\Arabidopsis_thaliana\GeneDiffExp\Pathway\CK-VS-24h.htm#gene10) | 34 (3.69)% | 409 (2.26)% | 0.003518728 | 0.037832947 | ko00940 |
| 11 | [Valine, leucine and isoleucine degradation](file:///E:\%E8%AF%95%E9%AA%8C%E7%BB%93%E6%9E%9C%E6%95%B0%E6%8D%AE\%E6%A0%B9%E8%82%BF%E4%BE%B5%E6%9F%93%E6%8B%9F%E5%8D%97%E8%8A%A5\%E6%8B%9F%E5%8D%97%E8%8A%A5%E5%AD%97%E8%A1%A8%E8%BE%BE%E8%B0%B1%E7%BB%93%E6%9E%9C%E6%95%B0%E6%8D%AE\%E8%A1%A8%E8%BE%BE%E8%B0%B1%E7%BB%93%E6%9E%9C\Arabidopsis_thaliana\GeneDiffExp\Pathway\CK-VS-24h.htm#gene11) | 12 (1.3)% | 97 (0.54)% | 0.003749211 | 0.037832947 | ko00280 |
| 12 | [Nitrogen metabolism](file:///E:\%E8%AF%95%E9%AA%8C%E7%BB%93%E6%9E%9C%E6%95%B0%E6%8D%AE\%E6%A0%B9%E8%82%BF%E4%BE%B5%E6%9F%93%E6%8B%9F%E5%8D%97%E8%8A%A5\%E6%8B%9F%E5%8D%97%E8%8A%A5%E5%AD%97%E8%A1%A8%E8%BE%BE%E8%B0%B1%E7%BB%93%E6%9E%9C%E6%95%B0%E6%8D%AE\%E8%A1%A8%E8%BE%BE%E8%B0%B1%E7%BB%93%E6%9E%9C\Arabidopsis_thaliana\GeneDiffExp\Pathway\CK-VS-24h.htm#gene12) | 12 (1.3)% | 105 (0.58)% | 0.007099527 | 0.065670625 | ko00910 |
| 13 | [Limonene and pinene degradation](file:///E:\%E8%AF%95%E9%AA%8C%E7%BB%93%E6%9E%9C%E6%95%B0%E6%8D%AE\%E6%A0%B9%E8%82%BF%E4%BE%B5%E6%9F%93%E6%8B%9F%E5%8D%97%E8%8A%A5\%E6%8B%9F%E5%8D%97%E8%8A%A5%E5%AD%97%E8%A1%A8%E8%BE%BE%E8%B0%B1%E7%BB%93%E6%9E%9C%E6%95%B0%E6%8D%AE\%E8%A1%A8%E8%BE%BE%E8%B0%B1%E7%BB%93%E6%9E%9C\Arabidopsis_thaliana\GeneDiffExp\Pathway\CK-VS-24h.htm#gene13) | 23 (2.5)% | 267 (1.48)% | 0.009845062 | 0.084061683 | ko00903 |
| 14 | [Pentose and glucuronate interconversions](file:///E:\%E8%AF%95%E9%AA%8C%E7%BB%93%E6%9E%9C%E6%95%B0%E6%8D%AE\%E6%A0%B9%E8%82%BF%E4%BE%B5%E6%9F%93%E6%8B%9F%E5%8D%97%E8%8A%A5\%E6%8B%9F%E5%8D%97%E8%8A%A5%E5%AD%97%E8%A1%A8%E8%BE%BE%E8%B0%B1%E7%BB%93%E6%9E%9C%E6%95%B0%E6%8D%AE\%E8%A1%A8%E8%BE%BE%E8%B0%B1%E7%BB%93%E6%9E%9C\Arabidopsis_thaliana\GeneDiffExp\Pathway\CK-VS-24h.htm#gene14) | 20 (2.17)% | 239 (1.32)% | 0.02036841 | 0.154027078 | ko00040 |
| 15 | [Metabolic pathways](file:///E:\%E8%AF%95%E9%AA%8C%E7%BB%93%E6%9E%9C%E6%95%B0%E6%8D%AE\%E6%A0%B9%E8%82%BF%E4%BE%B5%E6%9F%93%E6%8B%9F%E5%8D%97%E8%8A%A5\%E6%8B%9F%E5%8D%97%E8%8A%A5%E5%AD%97%E8%A1%A8%E8%BE%BE%E8%B0%B1%E7%BB%93%E6%9E%9C%E6%95%B0%E6%8D%AE\%E8%A1%A8%E8%BE%BE%E8%B0%B1%E7%BB%93%E6%9E%9C\Arabidopsis_thaliana\GeneDiffExp\Pathway\CK-VS-24h.htm#gene15) | 240 (26.06)% | 4199 (23.22)% | 0.02081447 | 0.154027078 | ko01100 |
| 16 | [Ascorbate and aldarate metabolism](file:///E:\%E8%AF%95%E9%AA%8C%E7%BB%93%E6%9E%9C%E6%95%B0%E6%8D%AE\%E6%A0%B9%E8%82%BF%E4%BE%B5%E6%9F%93%E6%8B%9F%E5%8D%97%E8%8A%A5\%E6%8B%9F%E5%8D%97%E8%8A%A5%E5%AD%97%E8%A1%A8%E8%BE%BE%E8%B0%B1%E7%BB%93%E6%9E%9C%E6%95%B0%E6%8D%AE\%E8%A1%A8%E8%BE%BE%E8%B0%B1%E7%BB%93%E6%9E%9C\Arabidopsis_thaliana\GeneDiffExp\Pathway\CK-VS-24h.htm#gene16) | 12 (1.3)% | 123 (0.68)% | 0.0229543 | 0.154323169 | ko00053 |
| 17 | [Plant hormone signal transduction](file:///E:\%E8%AF%95%E9%AA%8C%E7%BB%93%E6%9E%9C%E6%95%B0%E6%8D%AE\%E6%A0%B9%E8%82%BF%E4%BE%B5%E6%9F%93%E6%8B%9F%E5%8D%97%E8%8A%A5\%E6%8B%9F%E5%8D%97%E8%8A%A5%E5%AD%97%E8%A1%A8%E8%BE%BE%E8%B0%B1%E7%BB%93%E6%9E%9C%E6%95%B0%E6%8D%AE\%E8%A1%A8%E8%BE%BE%E8%B0%B1%E7%BB%93%E6%9E%9C\Arabidopsis_thaliana\GeneDiffExp\Pathway\CK-VS-24h.htm#gene17) | 33 (3.58)% | 451 (2.49)% | 0.02363508 | 0.154323169 | ko04075 |
| 18 | [Cysteine and methionine metabolism](file:///E:\%E8%AF%95%E9%AA%8C%E7%BB%93%E6%9E%9C%E6%95%B0%E6%8D%AE\%E6%A0%B9%E8%82%BF%E4%BE%B5%E6%9F%93%E6%8B%9F%E5%8D%97%E8%8A%A5\%E6%8B%9F%E5%8D%97%E8%8A%A5%E5%AD%97%E8%A1%A8%E8%BE%BE%E8%B0%B1%E7%BB%93%E6%9E%9C%E6%95%B0%E6%8D%AE\%E8%A1%A8%E8%BE%BE%E8%B0%B1%E7%BB%93%E6%9E%9C\Arabidopsis_thaliana\GeneDiffExp\Pathway\CK-VS-24h.htm#gene18) | 21 (2.28)% | 261 (1.44)% | 0.02613628 | 0.161173727 | ko00270 |
| 19 | [Glycerolipid metabolism](file:///E:\%E8%AF%95%E9%AA%8C%E7%BB%93%E6%9E%9C%E6%95%B0%E6%8D%AE\%E6%A0%B9%E8%82%BF%E4%BE%B5%E6%9F%93%E6%8B%9F%E5%8D%97%E8%8A%A5\%E6%8B%9F%E5%8D%97%E8%8A%A5%E5%AD%97%E8%A1%A8%E8%BE%BE%E8%B0%B1%E7%BB%93%E6%9E%9C%E6%95%B0%E6%8D%AE\%E8%A1%A8%E8%BE%BE%E8%B0%B1%E7%BB%93%E6%9E%9C\Arabidopsis_thaliana\GeneDiffExp\Pathway\CK-VS-24h.htm#gene19) | 12 (1.3)% | 130 (0.72)% | 0.0334587 | 0.191340135 | ko00561 |
| 20 | [Non-homologous end-joining](file:///E:\%E8%AF%95%E9%AA%8C%E7%BB%93%E6%9E%9C%E6%95%B0%E6%8D%AE\%E6%A0%B9%E8%82%BF%E4%BE%B5%E6%9F%93%E6%8B%9F%E5%8D%97%E8%8A%A5\%E6%8B%9F%E5%8D%97%E8%8A%A5%E5%AD%97%E8%A1%A8%E8%BE%BE%E8%B0%B1%E7%BB%93%E6%9E%9C%E6%95%B0%E6%8D%AE\%E8%A1%A8%E8%BE%BE%E8%B0%B1%E7%BB%93%E6%9E%9C\Arabidopsis_thaliana\GeneDiffExp\Pathway\CK-VS-24h.htm#gene20) | 5 (0.54)% | 36 (0.2)% | 0.03447570 | 0.191340135 | ko03450 |
| 21 | [alpha-Linolenic acid metabolism](file:///E:\%E8%AF%95%E9%AA%8C%E7%BB%93%E6%9E%9C%E6%95%B0%E6%8D%AE\%E6%A0%B9%E8%82%BF%E4%BE%B5%E6%9F%93%E6%8B%9F%E5%8D%97%E8%8A%A5\%E6%8B%9F%E5%8D%97%E8%8A%A5%E5%AD%97%E8%A1%A8%E8%BE%BE%E8%B0%B1%E7%BB%93%E6%9E%9C%E6%95%B0%E6%8D%AE\%E8%A1%A8%E8%BE%BE%E8%B0%B1%E7%BB%93%E6%9E%9C\Arabidopsis_thaliana\GeneDiffExp\Pathway\CK-VS-24h.htm#gene21) | 11 (1.19)% | 118 (0.65)% | 0.03821223 | 0.201978930 | ko00592 |
| 22 | [Phenylalanine metabolism](file:///E:\%E8%AF%95%E9%AA%8C%E7%BB%93%E6%9E%9C%E6%95%B0%E6%8D%AE\%E6%A0%B9%E8%82%BF%E4%BE%B5%E6%9F%93%E6%8B%9F%E5%8D%97%E8%8A%A5\%E6%8B%9F%E5%8D%97%E8%8A%A5%E5%AD%97%E8%A1%A8%E8%BE%BE%E8%B0%B1%E7%BB%93%E6%9E%9C%E6%95%B0%E6%8D%AE\%E8%A1%A8%E8%BE%BE%E8%B0%B1%E7%BB%93%E6%9E%9C\Arabidopsis_thaliana\GeneDiffExp\Pathway\CK-VS-24h.htm#gene22) | 16 (1.74)% | 196 (1.08)% | 0.04274877 | 0.205489952 | ko00360 |
| 23 | [Linoleic acid metabolism](file:///E:\%E8%AF%95%E9%AA%8C%E7%BB%93%E6%9E%9C%E6%95%B0%E6%8D%AE\%E6%A0%B9%E8%82%BF%E4%BE%B5%E6%9F%93%E6%8B%9F%E5%8D%97%E8%8A%A5\%E6%8B%9F%E5%8D%97%E8%8A%A5%E5%AD%97%E8%A1%A8%E8%BE%BE%E8%B0%B1%E7%BB%93%E6%9E%9C%E6%95%B0%E6%8D%AE\%E8%A1%A8%E8%BE%BE%E8%B0%B1%E7%BB%93%E6%9E%9C\Arabidopsis_thaliana\GeneDiffExp\Pathway\CK-VS-24h.htm#gene23) | 6 (0.65)% | 51 (0.28)% | 0.04400525 | 0.205489952 | ko00591 |
| 24 | [Glycolysis / Gluconeogenesis](file:///E:\%E8%AF%95%E9%AA%8C%E7%BB%93%E6%9E%9C%E6%95%B0%E6%8D%AE\%E6%A0%B9%E8%82%BF%E4%BE%B5%E6%9F%93%E6%8B%9F%E5%8D%97%E8%8A%A5\%E6%8B%9F%E5%8D%97%E8%8A%A5%E5%AD%97%E8%A1%A8%E8%BE%BE%E8%B0%B1%E7%BB%93%E6%9E%9C%E6%95%B0%E6%8D%AE\%E8%A1%A8%E8%BE%BE%E8%B0%B1%E7%BB%93%E6%9E%9C\Arabidopsis_thaliana\GeneDiffExp\Pathway\CK-VS-24h.htm#gene24) | 16 (1.74)% | 197 (1.09)% | 0.04443026 | 0.205489952 | ko00010 |

**Pathway (48h VS CK):**

| **#** | **Pathway** | **DEGs with pathway annotation (659)** | **All genes with pathway annotation (18086)** | **P value** | **Q value** | **Pathway ID** |
| --- | --- | --- | --- | --- | --- | --- |
| 1 | [Glucosinolate biosynthesis](file:///E:\%E8%AF%95%E9%AA%8C%E7%BB%93%E6%9E%9C%E6%95%B0%E6%8D%AE\%E6%A0%B9%E8%82%BF%E4%BE%B5%E6%9F%93%E6%8B%9F%E5%8D%97%E8%8A%A5\%E6%8B%9F%E5%8D%97%E8%8A%A5%E5%AD%97%E8%A1%A8%E8%BE%BE%E8%B0%B1%E7%BB%93%E6%9E%9C%E6%95%B0%E6%8D%AE\%E8%A1%A8%E8%BE%BE%E8%B0%B1%E7%BB%93%E6%9E%9C\Arabidopsis_thaliana\GeneDiffExp\Pathway\CK-VS-48h.htm#gene1) | 13 (1.97%) | 92 (0.51%) | 2.803224e-05 | 0.002971417 | ko00966 |
| 2 | [Tryptophan metabolism](file:///E:\%E8%AF%95%E9%AA%8C%E7%BB%93%E6%9E%9C%E6%95%B0%E6%8D%AE\%E6%A0%B9%E8%82%BF%E4%BE%B5%E6%9F%93%E6%8B%9F%E5%8D%97%E8%8A%A5\%E6%8B%9F%E5%8D%97%E8%8A%A5%E5%AD%97%E8%A1%A8%E8%BE%BE%E8%B0%B1%E7%BB%93%E6%9E%9C%E6%95%B0%E6%8D%AE\%E8%A1%A8%E8%BE%BE%E8%B0%B1%E7%BB%93%E6%9E%9C\Arabidopsis_thaliana\GeneDiffExp\Pathway\CK-VS-48h.htm#gene2) | 20 (3.03%) | 208 (1.15%) | 7.73874e-05 | 0.003181217 | ko00380 |
| 3 | [Circadian rhythm - plant](file:///E:\%E8%AF%95%E9%AA%8C%E7%BB%93%E6%9E%9C%E6%95%B0%E6%8D%AE\%E6%A0%B9%E8%82%BF%E4%BE%B5%E6%9F%93%E6%8B%9F%E5%8D%97%E8%8A%A5\%E6%8B%9F%E5%8D%97%E8%8A%A5%E5%AD%97%E8%A1%A8%E8%BE%BE%E8%B0%B1%E7%BB%93%E6%9E%9C%E6%95%B0%E6%8D%AE\%E8%A1%A8%E8%BE%BE%E8%B0%B1%E7%BB%93%E6%9E%9C\Arabidopsis_thaliana\GeneDiffExp\Pathway\CK-VS-48h.htm#gene3) | 27 (4.1%) | 332 (1.84%) | 9.003445e-05 | 0.003181217 | ko04712 |
| 4 | [Flavonoid biosynthesis](file:///E:\%E8%AF%95%E9%AA%8C%E7%BB%93%E6%9E%9C%E6%95%B0%E6%8D%AE\%E6%A0%B9%E8%82%BF%E4%BE%B5%E6%9F%93%E6%8B%9F%E5%8D%97%E8%8A%A5\%E6%8B%9F%E5%8D%97%E8%8A%A5%E5%AD%97%E8%A1%A8%E8%BE%BE%E8%B0%B1%E7%BB%93%E6%9E%9C%E6%95%B0%E6%8D%AE\%E8%A1%A8%E8%BE%BE%E8%B0%B1%E7%BB%93%E6%9E%9C\Arabidopsis_thaliana\GeneDiffExp\Pathway\CK-VS-48h.htm#gene4) | 25 (3.79%) | 302 (1.67%) | 0.0001228868 | 0.003256500 | ko00941 |
| 5 | [Biosynthesis of secondary metabolites](file:///E:\%E8%AF%95%E9%AA%8C%E7%BB%93%E6%9E%9C%E6%95%B0%E6%8D%AE\%E6%A0%B9%E8%82%BF%E4%BE%B5%E6%9F%93%E6%8B%9F%E5%8D%97%E8%8A%A5\%E6%8B%9F%E5%8D%97%E8%8A%A5%E5%AD%97%E8%A1%A8%E8%BE%BE%E8%B0%B1%E7%BB%93%E6%9E%9C%E6%95%B0%E6%8D%AE\%E8%A1%A8%E8%BE%BE%E8%B0%B1%E7%BB%93%E6%9E%9C\Arabidopsis_thaliana\GeneDiffExp\Pathway\CK-VS-48h.htm#gene5) | 119 (18.06%) | 2469 (13.65%) | 0.0007202186 | 0.014839823 | ko01110 |
| 6 | [Starch and sucrose metabolism](file:///E:\%E8%AF%95%E9%AA%8C%E7%BB%93%E6%9E%9C%E6%95%B0%E6%8D%AE\%E6%A0%B9%E8%82%BF%E4%BE%B5%E6%9F%93%E6%8B%9F%E5%8D%97%E8%8A%A5\%E6%8B%9F%E5%8D%97%E8%8A%A5%E5%AD%97%E8%A1%A8%E8%BE%BE%E8%B0%B1%E7%BB%93%E6%9E%9C%E6%95%B0%E6%8D%AE\%E8%A1%A8%E8%BE%BE%E8%B0%B1%E7%BB%93%E6%9E%9C\Arabidopsis_thaliana\GeneDiffExp\Pathway\CK-VS-48h.htm#gene6) | 32 (4.86%) | 482 (2.67%) | 0.00083999 | 0.014839823 | ko00500 |
| 7 | [Metabolic pathways](file:///E:\%E8%AF%95%E9%AA%8C%E7%BB%93%E6%9E%9C%E6%95%B0%E6%8D%AE\%E6%A0%B9%E8%82%BF%E4%BE%B5%E6%9F%93%E6%8B%9F%E5%8D%97%E8%8A%A5\%E6%8B%9F%E5%8D%97%E8%8A%A5%E5%AD%97%E8%A1%A8%E8%BE%BE%E8%B0%B1%E7%BB%93%E6%9E%9C%E6%95%B0%E6%8D%AE\%E8%A1%A8%E8%BE%BE%E8%B0%B1%E7%BB%93%E6%9E%9C\Arabidopsis_thaliana\GeneDiffExp\Pathway\CK-VS-48h.htm#gene7) | 184 (27.92%) | 4199 (23.22%) | 0.002431006 | 0.036812377 | ko01100 |
| 8 | [Valine, leucine and isoleucine degradation](file:///E:\%E8%AF%95%E9%AA%8C%E7%BB%93%E6%9E%9C%E6%95%B0%E6%8D%AE\%E6%A0%B9%E8%82%BF%E4%BE%B5%E6%9F%93%E6%8B%9F%E5%8D%97%E8%8A%A5\%E6%8B%9F%E5%8D%97%E8%8A%A5%E5%AD%97%E8%A1%A8%E8%BE%BE%E8%B0%B1%E7%BB%93%E6%9E%9C%E6%95%B0%E6%8D%AE\%E8%A1%A8%E8%BE%BE%E8%B0%B1%E7%BB%93%E6%9E%9C\Arabidopsis_thaliana\GeneDiffExp\Pathway\CK-VS-48h.htm#gene8) | 10 (1.52%) | 97 (0.54%) | 0.002802277 | 0.037130170 | ko00280 |
| 9 | [Cyanoamino acid metabolism](file:///E:\%E8%AF%95%E9%AA%8C%E7%BB%93%E6%9E%9C%E6%95%B0%E6%8D%AE\%E6%A0%B9%E8%82%BF%E4%BE%B5%E6%9F%93%E6%8B%9F%E5%8D%97%E8%8A%A5\%E6%8B%9F%E5%8D%97%E8%8A%A5%E5%AD%97%E8%A1%A8%E8%BE%BE%E8%B0%B1%E7%BB%93%E6%9E%9C%E6%95%B0%E6%8D%AE\%E8%A1%A8%E8%BE%BE%E8%B0%B1%E7%BB%93%E6%9E%9C\Arabidopsis_thaliana\GeneDiffExp\Pathway\CK-VS-48h.htm#gene9) | 13 (1.97%) | 154 (0.85%) | 0.004217123 | 0.048597502 | ko00460 |
| 10 | [Stilbenoid, diarylheptanoid and gingerol biosynthesis](file:///E:\%E8%AF%95%E9%AA%8C%E7%BB%93%E6%9E%9C%E6%95%B0%E6%8D%AE\%E6%A0%B9%E8%82%BF%E4%BE%B5%E6%9F%93%E6%8B%9F%E5%8D%97%E8%8A%A5\%E6%8B%9F%E5%8D%97%E8%8A%A5%E5%AD%97%E8%A1%A8%E8%BE%BE%E8%B0%B1%E7%BB%93%E6%9E%9C%E6%95%B0%E6%8D%AE\%E8%A1%A8%E8%BE%BE%E8%B0%B1%E7%BB%93%E6%9E%9C\Arabidopsis_thaliana\GeneDiffExp\Pathway\CK-VS-48h.htm#gene10) | 21 (3.19%) | 308 (1.7%) | 0.00458467 | 0.048597502 | ko00945 |
| 11 | [Alanine, aspartate and glutamate metabolism](file:///E:\%E8%AF%95%E9%AA%8C%E7%BB%93%E6%9E%9C%E6%95%B0%E6%8D%AE\%E6%A0%B9%E8%82%BF%E4%BE%B5%E6%9F%93%E6%8B%9F%E5%8D%97%E8%8A%A5\%E6%8B%9F%E5%8D%97%E8%8A%A5%E5%AD%97%E8%A1%A8%E8%BE%BE%E8%B0%B1%E7%BB%93%E6%9E%9C%E6%95%B0%E6%8D%AE\%E8%A1%A8%E8%BE%BE%E8%B0%B1%E7%BB%93%E6%9E%9C\Arabidopsis_thaliana\GeneDiffExp\Pathway\CK-VS-48h.htm#gene11) | 12 (1.82%) | 141 (0.78%) | 0.00548671 | 0.052871933 | ko00250 |
| 12 | [Cysteine and methionine metabolism](file:///E:\%E8%AF%95%E9%AA%8C%E7%BB%93%E6%9E%9C%E6%95%B0%E6%8D%AE\%E6%A0%B9%E8%82%BF%E4%BE%B5%E6%9F%93%E6%8B%9F%E5%8D%97%E8%8A%A5\%E6%8B%9F%E5%8D%97%E8%8A%A5%E5%AD%97%E8%A1%A8%E8%BE%BE%E8%B0%B1%E7%BB%93%E6%9E%9C%E6%95%B0%E6%8D%AE\%E8%A1%A8%E8%BE%BE%E8%B0%B1%E7%BB%93%E6%9E%9C\Arabidopsis_thaliana\GeneDiffExp\Pathway\CK-VS-48h.htm#gene12) | 18 (2.73%) | 261 (1.44%) | 0.007407973 | 0.065437095 | ko00270 |
| 13 | [Non-homologous end-joining](file:///E:\%E8%AF%95%E9%AA%8C%E7%BB%93%E6%9E%9C%E6%95%B0%E6%8D%AE\%E6%A0%B9%E8%82%BF%E4%BE%B5%E6%9F%93%E6%8B%9F%E5%8D%97%E8%8A%A5\%E6%8B%9F%E5%8D%97%E8%8A%A5%E5%AD%97%E8%A1%A8%E8%BE%BE%E8%B0%B1%E7%BB%93%E6%9E%9C%E6%95%B0%E6%8D%AE\%E8%A1%A8%E8%BE%BE%E8%B0%B1%E7%BB%93%E6%9E%9C\Arabidopsis_thaliana\GeneDiffExp\Pathway\CK-VS-48h.htm#gene13) | 5 (0.76%) | 36 (0.2%) | 0.009359788 | 0.076318271 | ko03450 |
| 14 | [Pentose and glucuronate interconversions](file:///E:\%E8%AF%95%E9%AA%8C%E7%BB%93%E6%9E%9C%E6%95%B0%E6%8D%AE\%E6%A0%B9%E8%82%BF%E4%BE%B5%E6%9F%93%E6%8B%9F%E5%8D%97%E8%8A%A5\%E6%8B%9F%E5%8D%97%E8%8A%A5%E5%AD%97%E8%A1%A8%E8%BE%BE%E8%B0%B1%E7%BB%93%E6%9E%9C%E6%95%B0%E6%8D%AE\%E8%A1%A8%E8%BE%BE%E8%B0%B1%E7%BB%93%E6%9E%9C\Arabidopsis_thaliana\GeneDiffExp\Pathway\CK-VS-48h.htm#gene14) | 16 (2.43%) | 239 (1.32%) | 0.01449273 | 0.101366276 | ko00040 |
| 15 | [Benzoxazinoid biosynthesis](file:///E:\%E8%AF%95%E9%AA%8C%E7%BB%93%E6%9E%9C%E6%95%B0%E6%8D%AE\%E6%A0%B9%E8%82%BF%E4%BE%B5%E6%9F%93%E6%8B%9F%E5%8D%97%E8%8A%A5\%E6%8B%9F%E5%8D%97%E8%8A%A5%E5%AD%97%E8%A1%A8%E8%BE%BE%E8%B0%B1%E7%BB%93%E6%9E%9C%E6%95%B0%E6%8D%AE\%E8%A1%A8%E8%BE%BE%E8%B0%B1%E7%BB%93%E6%9E%9C\Arabidopsis_thaliana\GeneDiffExp\Pathway\CK-VS-48h.htm#gene15) | 7 (1.06%) | 71 (0.39%) | 0.01463193 | 0.101366276 | ko00402 |
| 16 | [Phenylpropanoid biosynthesis](file:///E:\%E8%AF%95%E9%AA%8C%E7%BB%93%E6%9E%9C%E6%95%B0%E6%8D%AE\%E6%A0%B9%E8%82%BF%E4%BE%B5%E6%9F%93%E6%8B%9F%E5%8D%97%E8%8A%A5\%E6%8B%9F%E5%8D%97%E8%8A%A5%E5%AD%97%E8%A1%A8%E8%BE%BE%E8%B0%B1%E7%BB%93%E6%9E%9C%E6%95%B0%E6%8D%AE\%E8%A1%A8%E8%BE%BE%E8%B0%B1%E7%BB%93%E6%9E%9C\Arabidopsis_thaliana\GeneDiffExp\Pathway\CK-VS-48h.htm#gene16) | 24 (3.64%) | 409 (2.26%) | 0.01530057 | 0.101366276 | ko00940 |
| 17 | [alpha-Linolenic acid metabolism](file:///E:\%E8%AF%95%E9%AA%8C%E7%BB%93%E6%9E%9C%E6%95%B0%E6%8D%AE\%E6%A0%B9%E8%82%BF%E4%BE%B5%E6%9F%93%E6%8B%9F%E5%8D%97%E8%8A%A5\%E6%8B%9F%E5%8D%97%E8%8A%A5%E5%AD%97%E8%A1%A8%E8%BE%BE%E8%B0%B1%E7%BB%93%E6%9E%9C%E6%95%B0%E6%8D%AE\%E8%A1%A8%E8%BE%BE%E8%B0%B1%E7%BB%93%E6%9E%9C\Arabidopsis_thaliana\GeneDiffExp\Pathway\CK-VS-48h.htm#gene17) | 9 (1.37%) | 118 (0.65%) | 0.02856938 | 0.175632696 | ko00592 |
| 18 | [Flavone and flavonol biosynthesis](file:///E:\%E8%AF%95%E9%AA%8C%E7%BB%93%E6%9E%9C%E6%95%B0%E6%8D%AE\%E6%A0%B9%E8%82%BF%E4%BE%B5%E6%9F%93%E6%8B%9F%E5%8D%97%E8%8A%A5\%E6%8B%9F%E5%8D%97%E8%8A%A5%E5%AD%97%E8%A1%A8%E8%BE%BE%E8%B0%B1%E7%BB%93%E6%9E%9C%E6%95%B0%E6%8D%AE\%E8%A1%A8%E8%BE%BE%E8%B0%B1%E7%BB%93%E6%9E%9C\Arabidopsis_thaliana\GeneDiffExp\Pathway\CK-VS-48h.htm#gene18) | 7 (1.06%) | 82 (0.45%) | 0.02982442 | 0.175632696 | ko00944 |
| 19 | [Ascorbate and aldarate metabolism](file:///E:\%E8%AF%95%E9%AA%8C%E7%BB%93%E6%9E%9C%E6%95%B0%E6%8D%AE\%E6%A0%B9%E8%82%BF%E4%BE%B5%E6%9F%93%E6%8B%9F%E5%8D%97%E8%8A%A5\%E6%8B%9F%E5%8D%97%E8%8A%A5%E5%AD%97%E8%A1%A8%E8%BE%BE%E8%B0%B1%E7%BB%93%E6%9E%9C%E6%95%B0%E6%8D%AE\%E8%A1%A8%E8%BE%BE%E8%B0%B1%E7%BB%93%E6%9E%9C\Arabidopsis_thaliana\GeneDiffExp\Pathway\CK-VS-48h.htm#gene19) | 9 (1.37%) | 123 (0.68%) | 0.03594299 | 0.200524049 | ko00053 |

**Supplementary Table 5 Hormonal regulation related genes of DEGs (A: 24h and B: 48h)**

**Supplementary Table 6 PR proteins and some MYB transcription factors of DEGs (A: 24h and B: 48h)**
